# Global Financing Facility investments for vulnerable populations: content analysis regarding maternal and newborn health and stillbirths in 11 African countries, 2015 to 2019

**DOI:** 10.1080/16549716.2024.2329369

**Published:** 2024-07-05

**Authors:** Mary Kinney, Meghan Bruce Kumar, Issa Kaboré, Joël Kiendrébéogo, Peter Waiswa, Joy E. Lawn

**Affiliations:** a School of Public Health, University of the Western Cape, Cape Town, South Africa; b Department of Nursing, Midwifery and Health, Nothumbria University, Newcastle upon Tyne, UK; c Health Systems and Research Ethics, KEMRI-Wellcome Trust Programme, Nairobi, Kenya; d Operations Division, Research, Expertise and Training Department, Recherche Pour la Santé et le Développement (RESADE, Ouagadougou, Burkina Faso; e Department of Research, Expertise and Capacity Building, Research, Expertise and Training Department, Recherche Pour la Santé et le Développement (RESADE), Ouagadougou, Burkina Faso; f Department of Public Health, University Joseph Ki-Zerbo, Ouagadougou, Burkina Faso; g Heidelberg Institute of Global Health, Medical Faculty and University Hospital, Heidelberg University, Heidelberg, Germany; h Department of Public Health, Institute of Tropical Medicine, Antwerp, Belgium; i School of Public Health, Makerere University College of Health Sciences, Kampala, Uganda; j Department of Infectious Disease Epidemiology and International Health, London School of Hygiene & Tropical Medicine, London, UK

**Keywords:** Global Financing Facility for Women, Children and Adolescents: Examining National Priorities, Processes and Investments, Maternal, newborn, stillbirth, neonatal, pregnancy, health financing, policy analysis

## Abstract

**Background:**

The Global Financing Facility (GFF) was launched in 2015 to catalyse increased domestic and external financing for reproductive, maternal, newborn, child, adolescent health, and nutrition. Half of the deaths along this continuum are neonatal deaths, stillbirths or maternal deaths; yet these topics receive the least aid financing across the continuum.

**Objectives:**

To conduct a policy content analysis of maternal and newborn health (MNH), including stillbirths, in GFF country planning documents, and assess the mortality burden related to the investment.

**Methods:**

Content analysis was conducted on 24 GFF policy documents, investment cases and project appraisal documents (PADs), from 11 African countries. We used a systematic data extraction approach and applied a framework for analysis considering mindset, measures, and money for MNH interventions and mentions of mortality outcomes. We compared PAD investments to MNH-related deaths by country.

**Results:**

For these 11 countries, USD$1,894 million of new funds were allocated through the PADs, including USD$303 million (16%) from GFF. All documents had strong content on MNH, with particular focus on pregnancy and childbirth interventions. The investment cases commonly included comprehensive results frameworks, and PADs generally had less technical content and fewer indicators. Mortality outcomes were mentioned, especially for maternal. Stillbirths were rarely included as targets. Countries had differing approaches to funding descriptions. PAD allocations are commensurate with the burden.

**Conclusions:**

The GFF country plans present a promising start in addressing MNH. Emphasising links between investments and burden, explicitly including stillbirth, and highlighting high-impact packages, as appropriate, could potentially increase impact.

## Paper context

- **Main finding:** Maternal and newborn health care packages are strongly included in the Global Financing Facility policy documents for 11 African countries, especially regarding pregnancy and childbirth, though less for stillbirth, or postnatal care, or small and sick newborn care.
- **Added knowledge:** This study is the first independent content analysis of Global Financing Facility investment cases and related project appraisal documents, revealing mostly consistent content for maternal and newborn health across documents and overall correlation between national mortality burden and investments committed.
- **Global health impact for policy and action:** The Global Financing Facility have demonstrated promising initial investments for maternal and newborn health, although there are also missed opportunities for strengthening, especially for some neonatal high-impact packages and counting impact on stillbirths.

## Background

Reproductive, maternal, newborn, child, adolescent health (RMNCAH) require investment to reduce deaths, improve human capital and meet national goals and targets [1]. Half of the annual 8.6 million maternal, perinatal, child and adolescent deaths globally occur amongst stillborns (1.9 million) and neonates (2.3 million) [2]. Additionally, there were an estimated 0.29 million maternal deaths in 2020 [3]. For all three outcomes (maternal deaths, neonatal deaths and stillbirths) progress has been slower in the last decade [4]. With the added challenge of COVID-19-related disruptions of essential pregnancy, childbirth and newborn care, it is clear there more investment in needed in maternal and newborn health (MNH) and to prevent stillbirths [5].

Financing for MNH remains a major bottleneck for progress [6]. Overseas development aid analyses of 20 years shows global funding for RMNCAH overall at 15.6 billion in 2019, with a slight decrease, and neonatal deaths mentioned in less than 10% of disbursements and stillbirths in 0.003% [7,8]. Global and national movements, such as Every Newborn Action Plan (ENAP) (2014) and the Strategy to End Preventable Maternal Mortality (EPMM) (2015) [9,10], have served to support countries to set national targets, linked to Sustainable Development Goal (SDGs), prioritise interventions and monitor progress for MNH. Despite some progress [4], health financing has been identified as a major gap with the only 12 of 106 low- and middle income countries (LMICs) reporting fully funded national MNH plans [4].

The Global Financing Facility (GFF), a multi-stakeholder global partnership housed at the World Bank (WB), was established in 2015 to close resource gaps for women’s, children’s and adolescents’ health, including for MNH. GFF aims to support LMIC with catalytic financing and technical assistance through country-led approaches to priority setting, supported by domestic health financing. Since 2015, GFF has partnered with 36 LMIC and claims to have raised US$2 billion of new funds through their partnerships unlocking US$32 billion for women, children, and adolescent health in partner countries [11]. A growing body of work has begun to independently examine the investment of the GFF [12–16]. George and colleagues examined GFF documents for 11 countries regarding adolescent sexual and reproductive health, revealing differences between countries and between the two main GFF country policy documents available, the investment cases (ICs) and project appraisal documents (PADs) [17].

MNH is a priority for the GFF due to the high burden of mortality and morbidity around the time of birth and in the first month of life [18], yet globally there is lower aid financing for this period in the lifecourse than other areas along the RMNCH continuum as well as poor resource tracking of dedicated domestic financing [4,7]. In this paper, we set out to analyse how GFF country planning documents have included MNH, including stillbirths, and the alignment with mortality burden. This paper forms part of a Special Series aimed at understanding policy content and processes related to the GFF in recipient countries in an effort to promote accountability of and learning from this global health initiative. Kumar and colleagues present a more detailed assessment of how quality is included in these documents [19].

## Methods

This study was a descriptive content analysis study examining policy content of the country-level planning documents through which the GFF is operationalised. GFF country-level planning documents include: (1) ICs, which delineates national RMNCAH priorities; and (2) PADs, which are WB documents describing the supported project. The GFF contributions are grants within the PADs and serve as an additional contribution of the total financing linked to the project, which is supported by WB and sometimes other partners. Our study applied a standard framework and used word searches to consider content of GFF documents regarding MNH. We followed the four-phase READ approach for document analysis [20], with the following steps: (1) readying our materials, (2) extracting data, (3) analysing data and (4) distilling findings.

### READ step 1: readying the materials: country selection and context

To ready the material, we initially searched the GFF website country pages to download the publicly available GFF policy documents (ICs and PADs). Additionally, we interacted with the GFF secretariat at the start of the research project to identify additional documents available and refinement of our selection criteria of documents, excluding restructured PADs or second round ICs.

Beginning from the set of 36 countries included in the GFF funding scheme, we selected countries if:

1) they had both GFF policy documents (ICs and PADs) available online or through the secretariat by June 2023; 2) both their available GFF policy documents were dated between 2015 and 2019, representing the first wave of GFF investment in countries and pre-dating the COVID-19 pandemic in 2020 which led to a major shift in WB and domestic health financing priorities; and 3) they tracked progress and prioritised domestic investment into MNH through participation in the Countdown to 2030 collaboration in Phase 2 [21] or they were a priority country for Newborn Essential Solutions and Technologies, or NEST360 [22].

Based on these criteria, 11 countries were selected for analysis: Burkina Faso, Cote d’Ivoire, Ethiopia, Kenya, Liberia, Malawi, Mali, Nigeria, Senegal, Tanzania, and Uganda (Supplementary File 1). While all of these countries are from the sub-Saharan Africa, they different in population size, cultural context, and health system structure and approach to delivering MNH [23,24]. There were 24 documents included in total (11 ICs and 13 PADs) (Supplementary file 2). Nigeria was the only country with multiple PADs linked to the IC, which are distinguished in this paper by their titles: Nigeria State Health Investment Project (NSHIP), Healthcare provision fund project (HUWE), and Accelerating Nutrition Results (Nutrition).

### READ step 2: extracting the data

To extract the data, we developed, tested, and refined tools to describe and quantify the content of the documents related to the continuum of maternal and newborn healthcare: packages of interventions for pregnancy, childbirth, postnatal care (PNC), and small and sick newborn care. We also mentions of mortality outcomes for mothers, newborns, and stillbirths (Supplementary file 3). Search terms were tested in English and French (Table 1). In addition to assessing the content, we counted how many times specific intervention packages were included in the documents.

**Table 1.** Search terms by category with the content analysis framework for Mentions, Mindset, Measures and Money.

Concept category	Related search terms
**MENTIONS of mortality outcomes**	
Maternal mortality	maternal death, maternal mortality, MMR
Stillbirth	Stillb*, SBR, perinatal death, fetal death, still birth
Newborn mortality	Newborn death, new-born death, neonatal death, NMR
**MINDSET, MEASURES, MONEY: care packages along MNH continuum (thematic content analysis)**	
Pregnancy care	antenatal, ANC, PMTCT, abortion, pregnan* (pregnant; pregnancy)
Childbirth care	skilled birth attend, SBA, skilled attend, ‘delivery’~ EmOC, EmONC, EmNOC, obstetric, resus, perinatal^ ~ delivery in relation to childbirth ^ including related to MPDSR
Postnatal care	postnatal, PNC, breast, milk
Small and sick newborn care	preterm, pre-term, prem*, Kangaroo, KMC, ‘birth weight’ - looking for low birth weight, LBW, Sick and small newborn (small and sick *), neonatal infection, ‘sepsis’ - include only for newborn or neonatal sepsis
Related health system issues	quality, quality of care, quality assurance, midw*, referral, ‘family’ - looking for family-centred, ‘respect’ - looking for respectful care/disrespect&abuse

The extraction tool, related guidance and summary process were developed iteratively and collectively by the author team. The extraction template includes sections on maternal and newborn (including stillbirth) health interventions with subsets to describe the following components: definition and description of terms, where it is mentioned in the document (specific sections or throughout), how it was framed (e.g. integrated or separate area of investment), and how interventions were included and described. Five individuals were involved in the data extraction process (JAK, IK, YK, MBK, MVK), including pilot-testing the tools for one country to ensure inter-rater reliability and meeting on a regular basis during the data extraction process to discuss experiences and align efforts.

### READ steps 3&4: analysis and distillation

**Figure 1.**
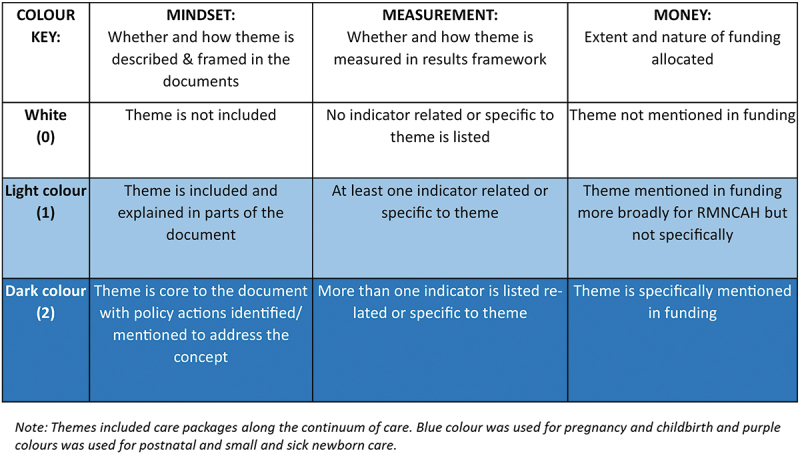
Framework for content analysis components: Mindset, Measures and Money.

To analyse the data, we entered the extracted data into a summary document using a standard template to capture key findings from each document and across documents for each country (ICs and PADs). We then considered the ‘M^3^ framework’ to examine mentions of MNH care packages in terms of content (mindset), indicators (measurement), and linked funding (money) [19]. Using the individual country summary documents, we applied a scoring system to each of the three items from the M^3^ framework (mindset, measurement, money) to grade the extent of inclusion and representation of packages of care along the MNH continuum of care: 1) pregnancy and childbirth, and 2) postnatal and small and sick newborn care (Figure 1).

To distil the data, we assigned colours to each score within the M^3^ frameworks to look for patterns within and between countries by document type. We considered the results by different contextual factors including level of institutional deliveries, mortality burdens (maternal, stillbirth, newborn), coverage of key MNH indicators (i.e. antenatal care (ANC), institutional deliveries, skilled birth attendance), and income (e.g. GDP per capita) [24]. Likewise, we sorted the results by the dates of publication for the policy documents, given the evolution of these key search terms in global health parlance and as in some countries (Bangladesh, Malawi, and Tanzania), the PADs pre-dated the ICs.

In addition, we counted the frequency of mentions of MNH-related mortality outcomes: maternal mortality, newborn mortality, and stillbirth. We applied a grading system whereby we assigned a document the colour green (best performance) if the document mentioned the mortality outcome more than once and included it as a target; orange if it mentioned the mortality outcome at least once but not as a target; and red if it did not mention the outcome at all.

For each country, we examined if there was consistency between the ICs and PADs in terms of MNH content priority to consider to see if the same interventions or strategies were included in both documents. To examine equity of investment linked to need, we compared the total value of investments described in all included PADs to the total burden of MNH-related deaths in 2015 using investment data from the PADs and mortality data from the UN estimates [2,3]. The total burden of deaths along the RMNCAH continuum of care in these 11 countries was 2.5 million in 2015, the year GFF launched, including maternal deaths, stillbirths, and death of children aged 0–24 years [2,3]. Of these deaths, 47% occurred due to complications in pregnancy, childbirth or in the postnatal period (comprising maternal, newborn deaths or stillbirths). We compared the mortality burden with the total value of the PAD, which is the full investment value of the project inclusive of the GFF grant, as well as only the total value of the GFF contribution to the PAD, which is a grant that contributes towards the project.

Analyses and findings were presented iteratively in two remote workshops (October 2021 and February 2022) and refined at in-person workshops (October 2022 and March 2023). Stakeholder engagement with the GFF Secretariat occurred at different points in the study, including meetings to present the research plan (July 2021) and to present and discuss preliminary results (May and June 2023) as well as multiple follow-up emails.

No ethical or special permissions were required for this study as it did not involve research on human subjects, and source documents are publicly available.

## Results

**Figure 2.**
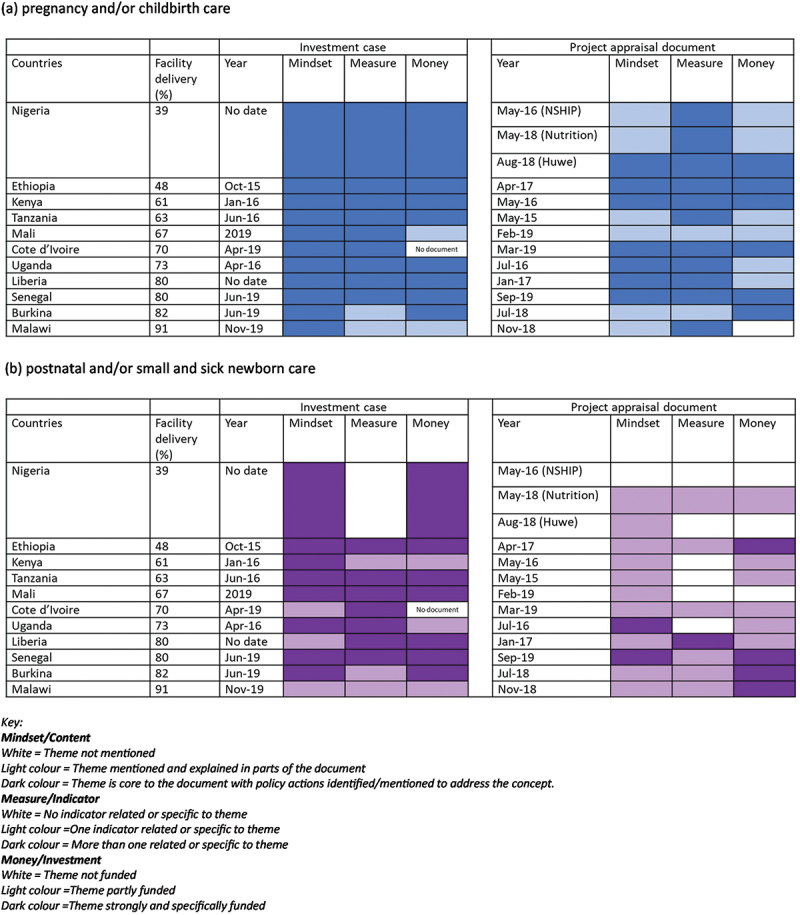
MNH policy content analysis across the continuum of care: mindset, measures and money (a) pregnancy and/or childbirth care (b) postnatal and small and sick newborn. Themes included care packages along the continuum of care. Blue colour was used for pregnancy and childbirth and purple colours was used for postnatal and small and sick newborn care.

Our content analysis reveals that MNH was included in all 24 GFF documents in some form, most commonly as an integrated concept and with more attention on improving, tracking and funding interventions during pregnancy and at birth. Figure 2 shows the grading of MNH packages of care across the country documents for the three components of the framework: mindset, measures and money. More details by country are available in Supplementary file 4 (Table S4.1).

### Interventions: content analysis

#### Mindset

MNH content commonly reflected the continuum of care approach integrating maternal and newborn issues together and as a core component of health system strengthening. Overall, there were specific sections on maternal health and often newborn health in the background section of the ICs. MNH-related interventions were included with more attention on care in pregnancy, notably ANC, and around the time of birth, i.e. emergency obstetric and newborn care (EmONC). In most PADs, MNH content focused primarily on care around the time of birth, notably strengthening access to EmONC and quality of care in pregnancy and childbirth. Despite the focus on pregnancy and childbirth, stillbirths, which are the highest impact outcome from pregnancy and childbirth care, were rarely mentioned (Box 1). Some country documents explicitly included strengthening maternal and perinatal death surveillance and response components as part of their PADs (Burkina Faso, Cote d’Ivoire, Kenya, Liberia, and Uganda).

**Box 1.** Stillbirth content.

For the most part, the GFF country documents rarely mention stillbirths specifically with few exceptions. All ICs include at least one mention of the related search terms; though these ranges from only one mention (e.g. Côte d’Ivoire, Ethiopia, Mali, Malawi, Senegal) to 36 mentions (e.g. Tanzania). Burkina Faso does not mention stillbirth as an outcome but as it relates to Civil Registration and Vital Statistics. Stillbirth is included as a mortality target only in one country (Tanzania) and is included in the results framework in six ICs (Ethiopia, Liberia, Kenya, Senegal, Uganda, Tanzania). The PADs mentions stillbirth even less with four countries not including it at all (Burkina Faso, Ethiopia, Mali, and Nigeria – all three PADs), and if it is mentioned, it is only once or few times as part of the background (Malawi, Liberia, Senegal) or part of maternal and perinatal audit (Kenya, Uganda, Cote d’Ivoire). Two exceptions include Cote d’Ivoire’s PAD, which describes a pilot project for mobile ultrasound clinic to identify high-risk pregnancies through fetal heartrate monitoring and reduce perinatal mortality and morbidity, and Tanzania’s PAD, which includes it as part of capturing maternal and perinatal deaths that take place in the community.

Documents also included content on postnatal care, but not strongly or frequently. Content on small and sick newborn care was more limited overall, though varied by country with some specific inclusion, mostly relating to Kangaroo Mother Care, neonatal infection and cord care. Small and sick newborn care was entirely absent in four country PADs (Burkina Faso, Ethiopia, Liberia, Nigeria-HUWE), and not included beyond one mention in Mali, Nigeria-Nutrition, and Senegal. Within each package of care, there were specific content consistently weak or missing across countries and documents including abortion care, fetal heart rate monitoring, respectful maternity or family-centered care, support for early initiation of breastfeeding, and midwifery.

Counts of mentions of interventions mirrored the policy content analysis showing more mentions of intervention packages for pregnancy and childbirth than for PNC, with few mentions of interventions specific to small and sick newborn care (Supplementary file 4, Table S4.2).

#### Measures

The resulting frameworks in most ICs had standard metrics aligned to ENAP/EPMM and included multiple MNH-related indicators, commonly ANC one or four visits, stillbirth birth attendance (SBA), and PNC within 2 days. The ICs contained comprehensive results frameworks that included broader health system indicators in addition to MNH mortality and coverage indicators. The PADs tended to have fewer indicators and often only included one or two MNH-related indicators (Supplementary file 4). All of the PADs had at least one maternal health indicator, notably ANC, SBA and/or EmONC. In cases, where the PAD had a broader focus on health system strengthening, indicators linked to pregnancy and childbirth were included over postnatal or small and sick newborn indicators. For example, Kenya’s PAD only included ANC and SBA as Program Development Objective (PDO) indicators even though the project focused overall on primary health care and quality of care including specific newborn care interventions, such as umbilical cord care. Five country PADs did not include any indicators specific to postnatal or small and sick newborn care (Nigeria, Kenya, Mali, Cote d’Ivoire and Uganda). Ethiopia’s PAD included developing and implementing a PNC directive to improve quality of PNC services in acknowledgement that the governance structure was needed to advance the intervention package. Burkina Faso’s PAD only had one MNH indicator, which is related to civil registration rather than health interventions (proportion of newborns receiving birth certificate). Indicators related to small and sick newborn care (e.g. Kangaroo Mother Care coverage or neonatal sepsis management) were found in some country ICs (i.e. Ethiopia and Mali) but in no PADs. There were also no indicators on respectful maternity care or family-centred care in any document.

#### Money

Differing approaches to funding (grant/loan/co-financing) and linked descriptions prevented meaningful comparisons of MNH-specific budgets and allocations (Supplementary file 4). The PADs provided a more common approach to financial documentation and totalled to USD$1,894 million overall across 11 countries (US$303 million from GFF specifically). For documents that did provide specific MNH content, allocation across countries and between countries were greatly inconsistent. The allocation for MNH in ICs ranged from 2.64% in Senegal to 50% in Kenya and in PADs from 2.3% in Tanzania to 100% in Senegal. There was also little consistency between country documents in terms of funding. For example, all ICs available specifically included descriptions relating to interventions in pregnancy and childbirth in the budgeting section; however, the same level of attention did not translate to funding descriptions in the PADs. For postnatal and newborn care, there was also less description in the PADs, and two countries did not include these at all in their funding descriptions (Nigeria – two PADs; Kenya).

### Consistency of content by country

Our mapping of the MNH content within each country found most documents to be consistent in focus areas or at least partly consistent related to MNH content (Table 2, Supplementary file 4). However, the focus on MNH diminished in most cases between a country’s IC and PAD. Intervention packages for care in pregnancy and at birth were included strongly in the ICs but content reduced in the PADs. Intervention packages for PNC were included to some variable degree in the ICs, but also reduced in the PADs. This disconnect was most evident in PADs, which often focused on broad structural improvements rather than specific focus on MNH.

**Table 2.** Consistency of MNH content in country documents.

Country	Consistency of MNH content	Short description of MNH content
Burkina Faso	Consistent	MNH integrated throughout both documents with inclusion of targets and core coverage indicators, and specific funding allocation. PAD takes more holistic health system strengthening approach for RMNCAH with focus on EmONC for MNH.
Cote d’Ivoire	Consistent	MNH included within both documents focusing more on pregnancy and childbirth interventions.
Ethiopia	Partially consistent	MNH included with related targets and indicators as well as budgets/allocations to related interventions.
Kenya	Partially consistent	MNH included in both documents; however, PAD has narrower focus on PHC and quality improvement and regulations.
Liberia	Consistent	MNH integrated throughout both documents. Core MNH indicators are included and partly consistent although ANC absent in PAD.
Malawi	Partially consistent	MNH embedded as part of health system strengthening in the IC; however, the PAD focuses on one component within, ECD.
Mali	Partially consistent	MNH embedded strongly with specific sections and indicators in IC; the PAD only includes it as part of the broader continuum with one indicator (ANC4)
Nigeria	Partially consistent	Unique example since country has multiple PADs. Nigeria’s IC includes MNH with clear attention to maternal health and some inclusion of newborn health interventions and mentions. The 3 PADs range in focus (nutrition, primary health care, and basic health provisions including MNH services – mostly care in pregnancy and at birth).
Senegal	Partially consistent	Broad health system approach with some links to MNH interventions as well as other areas along continuum, esp adolescent health.
Tanzania	Partially consistent	Documents differ in scope and content; the PAD precedes the investment case by a year MNH integrated with focus on EmONC in the IC and broader health system strengthening in the PAD.
Uganda	Consistent	Documents related and take a holistic approach to addressing RMNCAH. More mention of interventions during pregnancy and childbirth.

### MNH outcomes: content analysis

**Figure 3.**
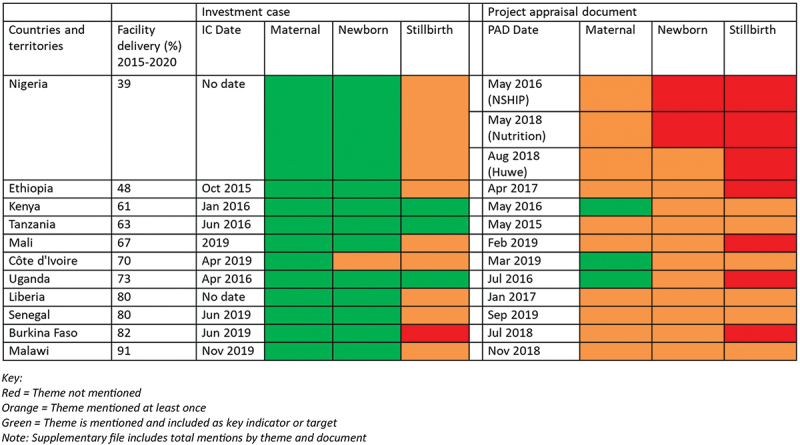
Mentions of the three outcomes (maternal, stillbirths and newborns) in GFF country planning documents.

The GFF policy documents contained content on maternal and newborn mortality outcomes with variation across countries and between country documents; mention of stillbirth was more limited and inconsistent (Figure 3). In terms of the frequency of mentions across the documents, maternal mortality was mentioned 1.7 times more than newborn mortality in the ICs and 2.3 times more in the PADs (Supplementary file 4, Table S4.4). For every one mention of stillbirth, there was at least four mentions of newborn mortality and seven mentions of maternal mortality. Overall, the PADs mentioned MNH outcomes less frequently than in the ICs. All ICs included specific mortality reduction targets for maternal mortality rate and 10 included neonatal mortality rate targets. Three PADs included maternal mortality as targets and all PADs mentioned it at least once. Newborn mortality was not included as a target in any PAD but was mentioned at least once in all but one country (two Nigeria PADs – NSHIP and Nutrition). Stillbirth was not mentioned at all in seven PADs.

#### Mortality burden compared to funding

**Figure 4.**
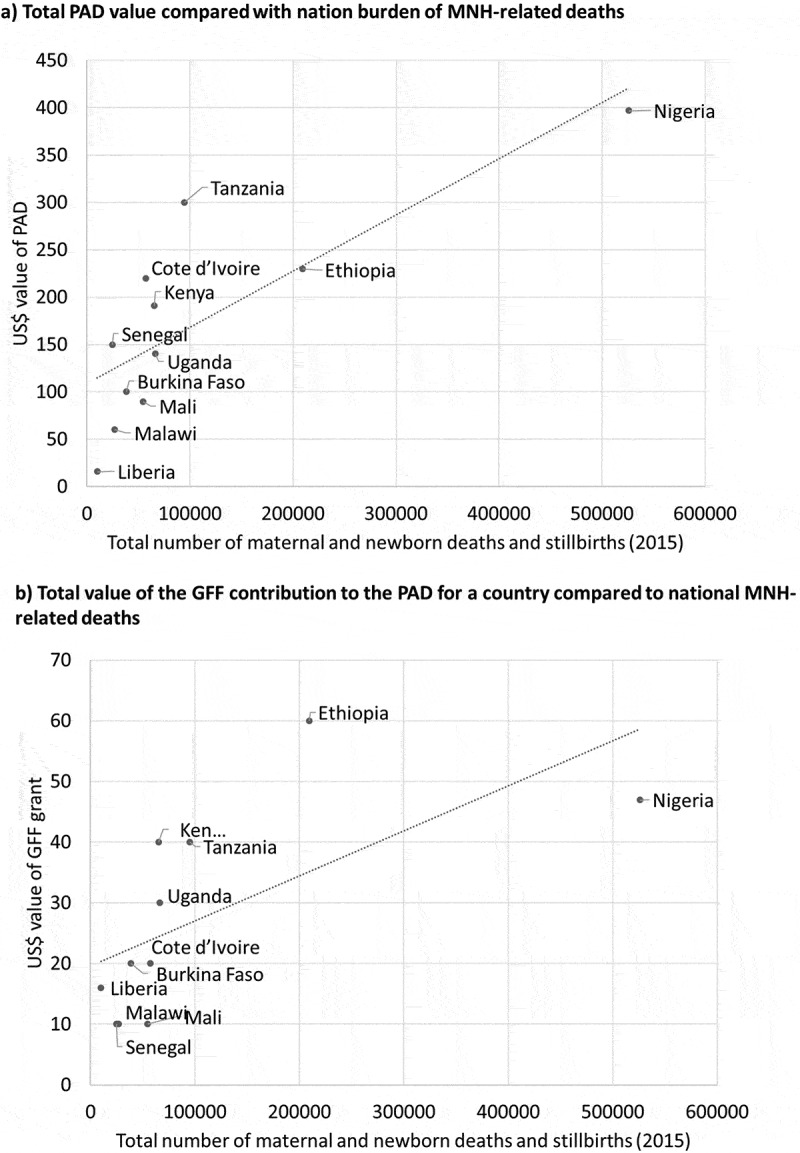
Comparison of GFF related investments to national maternal and neonatal deaths, plus stillbirths.

Across the 11 countries, the total investments made through the PADs encompassed USD$1,894 million of new funding from the WB towards the national ICs (USD$303 million from GFF grant) (Supplementary file – list of documents with years). Figure 4 compares the burden of these deaths by country to the total value of the countries’ PAD overall as well as to the specific GFF contribution to the PAD. Nigeria had the greatest burden of deaths and three PADs valued at USD 397 million in total. Liberia had the lowest burden of deaths and lowest PAD value (USD 16 million), although their PAD only included the GFF grant as an additional credit on the existing WB project. Tanzania’s PAD had the greatest value but not the greatest mortality burden. When considering the total value of the investments in relation to the total MNH mortality burden, the countries with the greatest burden received more funds. When comparing the investments with the mortality rates, there were no correlations (Supplementary File 4).

The PAD investment includes the GFF grant as well as other funding sources, such as The World Bank’s International Development Association (IDA) Data sources: PAD values and linked GFF contribution available in country PADs and presented in Supplementary File 4 (Table 4.5). Total number of neonatal deaths and stillbirths in 2015 accessed from UN IGME webpage [2] at childmortality.org. Total maternal deaths in 2015 accessed from UN report [3] available at http://www.who.int/publications/i/item/9789240068759

## Discussion

This study systematically analysed content in GFF policy documents related to MNH and stillbirths and is the first analysis in the public domain since the GFF started in 2015. By applying a standard policy analysis framework for 11 African countries including key word searches, we found strong focus on MNH overall with some specific gaps, notably stillbirth despite being a similar burden to neonatal deaths. Some packages of care within the MNH continuum of care were strongly reflected, such as pregnancy and childbirth; others less so, notably small and sick newborn care. Content, including indicators, was generally more restricted in the PADs than the ICs but varied between countries; however, MNH content was mostly consistent across the documents, and we observed an overall correlation between burden and investment. While the GFF may have advanced since these initial documents were developed, our study still has important implications for priority setting for MNH investments.

Maternal mortality and health were well articulated within the policy documents, which may be a legacy of the Safe Motherhood Initiative and a reflection of the first target for the SDG on health (SDG3.1 maternal survival) [25,26]. Newborn health packages were also included, to a lesser extent, reflecting efforts in the past decade to elevate the newborn in policy content, with a SDG target and clear packages of care [27,28]. The absence of stillbirths resonates with previous analyses looking at global and national policy documents [29,30] and financing [8]. Despite the availability of data, the current policy environment is less supportive for stillbirths as there is no SDG level target and this reflects in our findings. Acknowledging this gap, the GFF published a stillbirth roadmap in 2022 [31,32]. There remains a missed opportunity to better value the return on investments. Not counting stillbirths is undercounting impact, notably from antenatal and intrapartum interventions [4,33–35]. Initiatives seeking to bring the MNH and stillbirth agendas together, such as the combined ENAP and EPMM management structure [4], the Quality of Care Network [36] and AlignMNH (which organises an integrated MNH conference every other year) [37], may help shifts in future policy to include the full range of MNH mortality outcomes.

This study sheds light on the extent to which MNH issues are considered within the GFF country policy documents. Document analysis is commonly used in health policy research; and when done systematically and with rigour, it can be a helpful to inform the policy process [20,38]. Since policy documents are socially constructed outputs of a larger policy process [38–40], examining text within documents enables better understanding of the priorities, ideologies and beliefs of those who contribute to their development [41]. The content observed in these GFF country documents reflect the global MNH priorities at the time these documents were being formulated (2015–2019) and were mostly consistent between ICs and PADs. The first joint strategic objective from the global MNH strategies, ENAP and EPMM, was ‘strengthen care at birth’ [42], and this is a clear priority in the GFF country policy documents. However, the absence of stillbirth, including specific interventions, such as foetal heart rate monitoring [33], remains a concern; as does, the few mentions of critical health cadres, such as midwives.

As countries progress along the mortality transition model for maternal and neonatal mortality and stillbirth rates, the choice of priority interventions will adjust accordingly [43,44]. Countries with the highest mortality and most birth at home may focus more broadly on public health approaches to increase access whereas countries with lower levels of mortality need to shift to hospital care and quality of care, notably for small and sick newborns, without which the SDG3.2 cannot be met [44]. Most of the 11 countries included in this analysis started as mid-mortality settings [2,3], the PADs highlighted broader health systems strengthening investments over specific programmes for RMNCAH-N. By applying a diagonal approach, as demonstrated in Mexico [45], countries have the opportunity use vertical funding to build stronger health systems for MNH which is the cornerstone for stronger systems overall and also overcome damaging competition between issues [46]. The GFF offers a space to harmonise and align financing for RMNCAH-N, with smarter investments grounded in each country’s context [37].

Whilst pregnancy and childbirth care packages are clearly framed, PNC does not feature strongly in the documents and is a missed opportunity. PNC is important for a healthy start and for thriving as well as surviving though has a lower mortality impact [47]. The limited focus on small and sick newborn care may reflect that this package areas was still emerging as global priorities when these GFF processes were being undertaken [44]; we would hope to see some change in the further rounds of GFF funding given this is the highest impact package for neonatal survival. The World Health Organization (WHO) small and sick newborn care guidelines was released only in 2020 [48]. Abortion care has political sensitivities and even though services may be provided; it is often not explicitly mentioned in these policy documents. MNH quality guidelines, which included experience of care, were released in 2016 [49], and are also not fully reflected.

ICs commonly included comprehensive results frameworks, inclusive of a range of MNH indicators; the PADs tended to include fewer indicators and focused on care in pregnancy and childbirth. Even though the PADs may have limited space for clinical or technical indicators, there is a risk of these projects being interpreted primarily as hardware and infrastructure investments rather than aligning more clearly with the more comprehensive ICs. It would help to have clear intentional framing around the degree of priority for different indicators in these documents. There is also a need to harmonize MNH metrics agendas and provided more harmonized technical support to countries when they develop related policy documents. WHO set up a technical advisory group to do this in 2017 [50], with some promise towards a joint list of priority indicators [4]. Country context must also be a consideration when selecting priority indicators.

### Strengths and limitations

Our analyses cover country-level documents developed in the first five years of the GFF, and before the COVID-19 pandemic. The global health landscape has shifted, with depleting attention on and resources for core health services, such as MNH [51]. At the same time, the GFF has been responsive to country and partner needs, with some rapid internal shifts and learning platforms established [52]. For content analysis of GFF country documents, it is difficult to conduct meaningful comparisons in part because of lack of clear guidance on what makes a good IC, how this might effectively catalyse investment, and how to measure that impact. There are opportunities for the addition of a planning checklist as a guide, or a reporting template to improve evidence-based financing in this area. The other options are for all plans to be peer reviewed by experts before they are finalised or to make the process more transparent and inclusive. Also, the PADs only describe one project that contributes to the IC, and more work and mapping needs to be done to investigate the full contribution [19]. Assessing the money component of the framework was especially challenging since the GFF policy documents do not have a standard approach for planned resource allocations across the RMNCAH continuum. Additionally, there is no one data source, which tracks the budget lines for RMNCAH across these countries. WHO works with countries to track some indicators on domestic expenditure on health [53], and the GFF has worked with countries to track national and donor resources for the ICs [54], but do not disaggregate the data or present their methodology. It was beyond the remit of this content analysis study to try to track domestic resources for MNH, but it is a noted gap in the literature.

This research provides the first known analysis of MNH content in GFF documents, using a systematic selection of countries validated with members of the GFF Secretariat to ensure comprehensive availability of relevant documents. We ensured consistency and replicability of the approach by using a common framework with other policy content analyses for the GFF documents [19], standard data extraction templates with search terms (Supplementary files), collective and iterative abstraction, and searchers who are native English and French speakers. While this was an independent study that was not commissioned, we engaged relevant global stakeholders throughout the process and considered their feedback on the country inclusion criteria, search terms and methodology, and the presentation and interpretation of results. We recommend additional research to understand the national level stakeholder perspectives on the inclusion of MNH, including stillbirths, in policy processes, such as the GFF.

The GFF country documents form one part of the policy process, which is complex and nonlinear [38], and represent content written at one point in time. There were limitations both of what is publicly available and limitations of documentation. Although these documents propose priority actions and plans for implementation and monitoring over the implementation period, they do not capture implementation or policy adaption. Additionally, the scoring system of the Ms framework includes some subjectivity in terms of determining the extent to which a theme was described. The use of standard tools and collective abstraction with multiple workshops to analyse the results was undertaken to mitigate this. We had to revisit some of the data collection and analysis process and made some modifications in response to the data. For example, we had initially extracted the results by maternal health and newborn health (Supplementary File 3); however, we found that this approach did not allow consideration of interventions that serve both the mother and the baby. It also made it difficult to understand what content was included relating to stillbirth. Therefore, we adjusted the analysis to present results by intervention packages and would recommend revisions to the tools applied to separate out stillbirths.

Despite some differences between countries and over time, there are many similarities to draw from for programmes and research. First, all of these countries do need to improve inclusion of stillbirth specifically, given the high burden of deaths, and stagnant progress despite preventability and the enormous losses to families and economies. Further work is needed to include stillbirth in policy documents as measurable, and actionable yet not just automatic from antenatal and intrapartum care since specific interventions such as syphilis screening, growth monitoring and fetal heart rate monitoring are needed for impact [29]. This recommendation is not just for GFF but more broadly for the MNH and global health community given consistent gaps in funding and action over the last decade [8,30,55]. Second, these countries require more investment overall in maternal and newborn care to reach the SDGs for reducing mortality. Many new and old interventions show promise for saving lives [56], but it will require political will with linked investment to implement them at scale and with quality. The assumption that investing in maternal health will automatically improve newborn survival or stillbirths is not evidence-based [57]. Specific interventions are needed for both. Lastly, learning from the content analyses included in this Special Series [19], more research is needed to assess power at the table for GFF and other investment processes at global and national levels.

## Conclusions

The GFF presents a real opportunity for MNH and these GFF country planning documents from 2015 to 2019 do reflect this as a major issue. Whilst MNH account for the majority of deaths in the GFF remit of RMNCAH-N plans, this study has uncovered gaps and opportunities to strengthen this MNH focus and better align burden with investments, particularly for stillbirths, and also for programmes beyond care at birth, importantly neonatal care. The MNH content in the GFF documents resonate with analyses of foreign aid and research priorities, with more priority for maternal mortality and related health interventions, some for neonatal and as yet little inclusion of stillbirth. As countries make progress and transition from higher mortality settings, different priorities will need to be considered to respond and keep improving outcomes and advancing health systems further. A checklist for planning or a standard reporting template could be useful to inform and track evidence-based financing in GFF for MNH and other topics. GFF is the main global health funding initiative for RMNCAH-N, and has an important role of engage relevant actors in their policy processes, including affected communities, using evidence and data, to inform their plans and make the strongest case for return on investment to then drive more yet investment and more progress.

## Supplementary Material

Supplemental Material

## Data Availability

The datasets used and/or analysed in this study are available from the corresponding author on reasonable request. This paper is part of the *Global Health Action* Special Series, “Global Financing Facility for Women, Children and Adolescents: Examining National Priorities, Processes and Investments”, available in Volume 17-01.
