# Supplementary material for: Global Financing Facility investments for vulnerable populations: content analysis regarding maternal and newborn health and stillbirths in 11 African countries, 2015 to 2019
Source: Glob Health Action. 2024 Jul 5;17(1):2329369. doi: 10.1080/16549716.2024.2329369 (PMC11229757; doi:10.1080/16549716.2024.2329369)
Supplement: Supplemental Material [file ZGHA_A_2329369_SM6256.pdf]

SUPPLEMENTARY FILES

Paper Title: Global Financing Facility investments for vulnerable populations: content analysis regarding maternal and newborn health and stillbirths in 11 African countries 2015 to 2019

Contents

Supplementary File 1. Country and document selection criteria and process for GFF MNH content analyses 2

Supplementary File 2. Documents included in analysis 3

Supplementary File 3. Extraction tools 6

GFF MNH Data Extraction Process per country: basic steps 6

GFF MNH analysis data extraction template 8

GFF MNH analysis country summary template 12

GFF MNH analysis - template for counting mentions of key concepts and words 14

GFF MNH analysis - template for counting mentions of mortality 17

Supplementary File 4. Detailed results 18

Table 4.1: Justification of scores by country by document 18

Table 4.2: Results for mentions of interventions packages in the MNH continuum of care 34

Table 4.3: Summary results by country, by document, by framework component 34

Table 4.4: Frequency of mentions 41

a) Frequency of mortality outcomes mentioned 41

b) Frequency of search terms mentioned 42

Supplementary File 1. Country and document selection criteria and process for GFF MNH content analyses

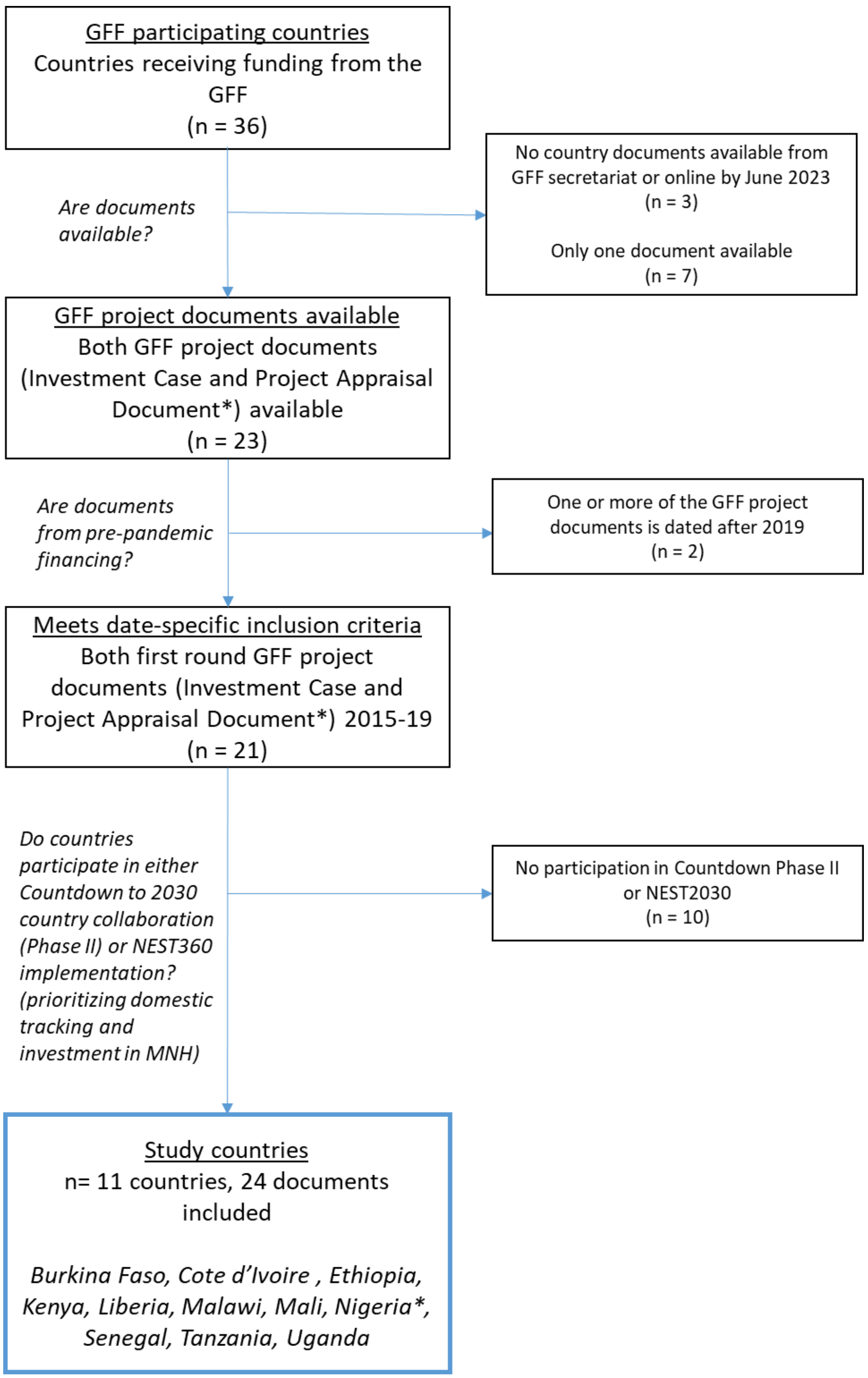

Supplementary File 2. Documents included in analysis

Table S2:1: Documents included in the content analysis

Countries	IC year	IC title	PAD year	PAD title	Weblinks
Burkina Faso	June 2019	AMELIORER LA SANTE DE LA REPRODUCTION, DE LA MERE, DU NOUVEAU-NE, DE L'ENFANT ET DE L'ADOLESCENT-JEUNE, DE LA NUTRITION ET DE L'ETAT CIVIL ET STATISTIQUES VITALES DOSSIER D'INVESTISSEMENT	July 2018	Health services reinforcement project	IC https://www.globalfinancingfacility.org/sites/default/files/Burkina-Faso_Investment_Case_FR.pdf<br><br>PAD https://documents1.worldbank.org/curated/en/570511531107056406/pdf/BURKINA-FASO-PAD-1-06182018.pdf
Côte d'Ivoire	2019	DOSSIER D'INVESTISSEMENT POUR LA RÉDUCTION DE LA MORTALITÉ MATERNELLE, NÉONATALE ET INFANTO-JUVENILE, ET POUR L'AMÉLIORATION DE LA SANTÉ DES ADOLESCENTS 2020-2022	March 2019	Strategic purchasing and alignment of resources and knowledge in health project (SPARK-HEALTH)	IC https://www.globalfinancingfacility.org/sites/gff_new/files/documents/Central-African-Republic-Investment-Case-resume-Fr.pdf<br><br>PAD https://documents1.worldbank.org/curated/en/242801553565658750/pdf/Côte-d-Ivoire-Strategic-Purchasing-and-Alignment-of-Resources-and-Knowledge-in-Health-Project.pdf
Ethiopia	October 2015	HEALTH SECTOR TRANSFORMATION PLAN 2015/16-2019/20 (2008-2012 EFY)	April 2017	The health sustainable development goals program for results	IC [Document originally downloaded from GFF webpage but no longer available] https://www.globalfinancingfacility.org/sites/gff_new/files/Ethiopia-Investment-Case.pdf<br><br>Document can be now accessed at: https://extranet.who.int/nutrition/gina/sites/default/filesstore/ETH%202016%20Health%20Sector%20Transformation%20Plan.pdf<br><br>PAD https://documents1.worldbank.org/curated/en/228241618346428068/pdf/Disclosable-Restructuring-Paper-Health-Sustainable-Development-Goals-Program-for-Results-P123531.pdf
Kenya	January 2016	KENYA REPRODUCTIVE, MATERNAL, NEWBORN, CHILD AND ADOLESCENT	May 2016	Transforming health systems for universal care project	IC https://www.globalfinancingfacility.org/sites/gff_new/files/Kenya-Investment-Case.pdf

		HEALTH (RMNCAH) INVESTMENT FRAMEWORK			PAD https://documents1.worldbank.org/curated/en/215261467995371106/pdf/PAD1694-PAD-P152394-IDA-R2016-0122-1-Box396259B-OUO-9.pdf
Liberia	No date	INVESTMENT CASE FOR REPRODUCTIVE, MATERNAL, NEW-BORN, CHILD, AND ADOLESCENT HEALTH 2016–2020	January 2017	Health systems strengthening project (additional credit)	IC https://www.globalfinancingfacility.org/sites/default/files/Liberia-Investment-Case.pdf<br><br>PAD https://documents1.worldbank.org/curated/en/473661488078030716/pdf/Liberia-RP-no-AF-02032017.pdf
Malawi	November 2019	THE GOVERNMENT OF MALAWI'S INVESTMENT CASE FOR REPRODUCTIVE, MATERNAL, NEWBORN, CHILD AND ADOLESCENT HEALTH AND NUTRITION 2020-2022	November 2018	Investing in early years for growth and projectivity in Malawi project	IC [Document originally provided by the GFF Secretariat in October 2023[https://www.globalfinancingfacility.org/sites/gff_new/files/documents/Malawi-GFF-Investment-Case.pdf<br><br>PAD https://documents1.worldbank.org/curated/en/172701545534083794/pdf/MALAWI-PADf-11302018-636811128679250963.pdf
Mali	2019	DOSSIER D'INVESTISSEMENT DE LA SANTE DE LA REPRODUCTION, DE LA MERE, DU NOUVEAU-NE, DE L'ADOLESCENT ET DE LA NUTRITION (SRMNEA+N 2019 -2023)	February 2019	Mali accelerating progress towards universal health coverage project	IC https://www.globalfinancingfacility.org/sites/gff_new/files/documents/Dossier-investment-de-la-SRMNEA-N_Mali.pdf<br><br>PAD https://documents1.worldbank.org/curated/en/382171551732681185/pdf/mali-pad-02282019-636871609042094203.pdf
Nigeria	No date	2017-2030 REPRODUCTIVE, MATERNAL, NEWBORN, CHILD, ADOLESCENT HEALTH AND NUTRITION	May 2016, May 2018, April 2018	PAD NSHIP- Nigeria State Health Investment Project dated May 2016 (additional credit)<br>PAD HUWE -- Healthcare provision fund project (Huwe project) dated August 2018<br>PAD Nutrition- Accelerating Nutrition	IC https://www.globalfinancingfacility.org/sites/default/files/2022-12/Nigeria-Investment-Case.pdf<br><br>PAD-NSHIP: *https://documents1.worldbank.org/curated/en/380321468179100435/pdf/PAD1741-PJPR-P120977-P157977-IDA-R2016-0125-1-Box396259B-OUO-9.pdf*<br><br>PAD-HUWE: *Document provided by the GFF Secretariat*<br><br>PAD Nutrition: *https://documents1.worldbank.org/curated/en/910491530329489994/pdf/NIGERIA-NUTRITION-PAD-05252018.pdf*

				results in Nigeria dated May 2018	
Senegal	2019	RÉDUCTION<br>DE LA MORTALITÉ MATERNELLE, NÉONATALE, INFANTO-JUVÉNILE,<br>DES ADOLESCENTS ET DES JEUNES<br>DOSSIER D'INVESTISSEMENT	September 2019	Investing in MCA health	IC<br>www.globalfinancingfacility.org/sites/gff_new/files/documents/Senegal-dossier-dinvestisement.pdf<br><br>PAD<br>https://documents1.worldbank.org/curated/en/469001569808857911/pdf/Senegal-Investing-in-Maternal-Child-and-Adolescent-Health-Project.pdf
Tanzania	June 2016	THE NATIONAL ROAD MAP STRATEGIC PLAN TO IMPROVE REPRODUCTIVE, MATERNAL, NEWBORN, CHILD & ADOLESCENT HEALTH IN TANZANIA (2016 - 2020). ONE PLAN II	May 2015	Strengthening primary health care for results program	IC<br>www.globalfinancingfacility.org/sites/gff_new/files/Tanzania_One_Plan_II.pdf<br><br>PAD<br>https://documents1.worldbank.org/curated/en/243871468179947102/pdf/96274-PAD-P152736-IDA-R2015-0121-1-Box391433B-OUO-9.pdf
Uganda	April 2016	INVESTMENT CASE FOR REPRODUCTIVE, MATERNAL, NEWBORN, CHILD AND ADOLESCENT HEALTH SHARPENED PLAN FOR UGANDA 2016/17-2019/20	July 2016	RMNCH services improvement project	IC<br>www.globalfinancingfacility.org/sites/default/files/Uganda-Investment-Case.pdf<br>PAD<br>https://documents1.worldbank.org/curated/en/854971471534008736/pdf/PAD-07182016.pdf

Source: Documents originally downloaded in July 2021 or provided by the Secretariat June 2023. Weblinks provided were accessed on 24 January 2024.

Supplementary File 3. Extraction tools

GFF MNH Data Extraction Process per country: basic steps

Step 1	• Assess country documents and prepare data extraction tool<br>• Go to Dropbox -> GFF newborn analysis -> Country docs: Locate country folder with documents (IC/PAD) in folder<br>• Go to Dropbox -> GFF newborn analysis -> Analysis: Locate template documents in folder and see below instructions. Create a folder for the country files in this folder, if not yet done:<br>• Open the document “GFF MNH Data Extraction Template” file and save as the following Word documents in the country folder:<br>➢ Word document A: “Country name/ IC/ date of extraction” [DDMMYYYY] example: “GFF MNH Uganda / IC / 12042021<br>➢ Word document B: “Country name/ PAD / date of extraction” [DDMMYYYY] example: “GFF MNH Uganda / PAD / 12042021<br>• Open the document “GFF MNH Country Summary Template” file and save as the following Word document:<br>➢ Word document C: “GFF MNH Country name / Summary / date of entry [DDMMYYYY] example: “GFF MNH Uganda summary 12042021<br>• Open the document “Excel to extract data” and save as the following Excel file:<br>➢ Excel file: Word document C: “GFF MNH Country name / search terms / date of entry [DDMMYYYY] example: “GFF MNH Uganda search terms 12042021
Step 2	• Search for terms in the investment case and PAD<br>• Use “advanced search” in Adobe PDF) to search for terms in the excel<br>• Record the number of mentions and high level take aways in the excel. Save your work in the country folder in “Analysis” folder.
Step 3	• Complete Word Documents A and B for data extraction<br>• Use template and answer questions<br>• To support responses, use quotes and screen shots to verify information and include page numbers.<br>• Enter in summary for each point within numbered topic areas.<br>• Provide 3-5 bullet point summarizing information and main take aways
Step 4	• Complete Word Document C<br>• Main impressions/ signature or unique features/ strengths / weaknesses (5 bullet points max)<br>• Copy and paste relevant sections from the country data extraction documents<br>• Write summary statements to synthesize the information for each section/question<br>• Mindset: Consistency of framing and/or evolution in thinking<br>• Money: Include the costed plan proposed in the IC and the estimated need and value of the PAD and the GFF component of the PAD<br>• Measurement: Consistency of indictors

GFF MNH analysis data extraction template

Instructions:

- *Save file as: Country name/ IC or PAD / date of extraction" [DDMMYYYY]*
- *Complete data extraction by answering questions in each section and providing summary points or copying in text from document in bullet form to respond to the below questions*
    - *Include page numbers for content*
    - *Include screen shots of relevant tables*
- *To find the information, use the search terms in the excel file.*

Prepared by NAME
Date: DATE
Country: NAME
Document: INDICATE IC OR PAD

Provide details about the document:

- Title:
- Date of publication
- Total pages

Respond to the questions or statements for each section. Use quotes and screen shots to verify information and include page numbers. Enter in summary for each point within numbered topic areas. Provide 3-5 bullet point summarizing information and main take aways.

1. Newborn (search terms: Newborn, Neonat*, Stillb*, Perinatal)
    a. Definition and description
        i. Copy definition if available
    b. Count of total mentions (but not extract text) and mentions as part of acronym
    c. For each mention:
        i. Where is it in the document (Table of Contents, Foreword, Situation Analysis, Indicators, Budget, etc)
            1. Is it consistently mentioned throughout the document, or does attention narrow or disappear as you move through to operational details, budgets, indicators? How so or why not?
            2. Any budget details at all?

        ii. Is it mentioned mainly as part of a larger acronym/ as an ‘add on’ within other topics or are there independent sections/ detailed analyses and programmes specifically for newborns?
            1. If there are independent sections note what they are
    d. Framing: Is NB/SB integrated into maternal and/or child or brought out as a separate area for investment?
    e. Service delivery lens: what key interventions are included and how described, eg:
        i. Resuscitation
        ii. preterm
        iii. kangaroo
        iv. low birth weight
        v. “small and sick”
        vi. postnatal
        vii. breast*

- *Add summary of main take aways here*

2. Maternal (search terms: matern*)
    a. Definition and description
        i. Copy definition if available
    b. Count of total mentions (but not extract text) and mentions as part of acronym
    c. For each mention:
        i. Where is it in the document (Table of Contents, Foreword, Situation Analysis, Indicators, Budget, etc)
            1. Is it consistently mentioned throughout the document, or does attention narrow or disappear as you move through to operational details, budgets, indicators? How so or why not?
            2. Any budget details at all?
        ii. Is it mentioned mainly as part of a larger acronym/ as an ‘add on’ within other topics or are there independent sections/ detailed analyses and programmes specifically for maternal?
            1. If there are independent sections note what they are
    d. Framing: How is mother-baby dyad or family-centred care mentioned?
    e. Service delivery lens: what key interventions are included and how described, eg:
        i. Antenatal care (ANC)
        ii. Prevention of Mother-To-Child Transmission (PMTCT)

iii. skilled birth attend*
iv. obstetric / Emergency Obstetric Care (EmOC)
v. abortion

- *Add summary here in bullets*

3. Health systems: Enabling environment to effectively deliver services/interventions benefiting
    a. Framing: Provide high level observations of content (1-2 sentence). Which Health System Building Blocks are mentioned explicitly in relation to MNH?
        i. Financing: budgeting and Results Based Financing
        ii. Info/data: indicators and tracking progress
        iii. Human Resources:
        iv. Management/governance: Maternal and Perinatal Death Surveillance and Response (MPDSR)
        v. Community:
        vi. Commodities:
        vii. Service delivery – systems: referral, networks of care, quality
        viii. Private sector
        ix. Civil Registration and Vital System (CRVS)
        x. Universal Health Coverage

Add summary here in bullets

4. Quality (search terms: quality, respectful care, referral, MPDSR)
    a. Framing: Do the GFF documents mention quality?
        i. If so, how do they define quality?
        ii. If they define it, what are the different components of quality (e.g. technical quality, patient experience)
    b. (referral/networks): How are different levels and actors described? Are interactions described to provide high quality care? (provider-patient/family, provider-provider across levels of care, with communities)

Add summary here in bullets

5. What is the overall rationale of the document (IC or PAD)
    a. Is it holistic? Is it focused on one aspect e.g. nutrition specific

b. How does it address inequity generally? How does it address vulnerable populations relating to MNH?
c. Any other comments

Add summary here in bullets

GFF MNH analysis country summary template

Instructions:

- *Save file as: Country name/summary/ date of entry" [DDMMYYYY]*
- *Copy and paste relevant sections from the country data extraction documents*
- *Write summary statements to synthesize the information for each section/question*

Prepared by NAME
Date: DATE
Country: NAME
Document: INDICATE IC OR PAD

Provide details about the documents:

- Title:
- Date of publication
- Total pages

Main impressions/ signature or unique features/ strengths / weaknesses (5 bullet points max)

- ***ADD YOUR NOTES***

Summary bullets for each document by section (complete the table)
For each concept, add a bullet with sentence on each:
Mindset: How the concept was described in the document?
Money: What budget allocations were included as relevant to the concept?
Measurement: What targets and indicators were included as relevant to the concept?

	IC – main take aways	PAD – main take aways
Newborn	add here	add here
Maternal		
Health Systems		
Quality		
Overall		

Comparing the IC and PAD:

1. Mindset:
    - Was there consistency of framing and/or evolution in thinking for MNH?

2. Money:
    - Include what was costed for MNH in each document.
    - Was there a costed plan proposed in the IC? If so, did this reflect in the estimated need and value of the PAD?
    - Other observations about the costed plan or proposed budget?

3. Measurement:
    - List relevant MNH indicators included in both documents
    - Is there consistency of indictors between documents?
    - What type of results/ indicators are used for MNH (mortality, morbidity, coverage, social determinants)
    - Are there specific results based financing indicators for MNH?
    - Any other Monitoring and Evaluation (M&E) relevant for MNH?

4. Another other observations

GFF MNH analysis - template for counting mentions of key concepts and words

		IC		PAD	
**Terms**	**How to search**	**# identified**	**Main take away**	**# identified**	**Main take away**
**MATERNAL (*not family planning)**					
maternal	general				
MMR	exact				
mother	general				
**PREGNANCY INTERVENTIONS**					
antenatal	general				
ANC	exact				
PMTCT	exact				
abortion	general				
pregnan* (pregnant; pregnancy)	general				
**STILLBIRTH**					
Stillb*	general search				
fetal	general search				
still birth	general search				
**CHILDBIRTH CARE**					
skilled birth attend	general				
SBA	exact				
skilled attend	general				
"delivery"~	general				
EmOC	general				
EmONC	general				
EmNOC	general				
obstetric	general				
resus	general search				
Perinatal	general search				
**NEWBORN**					

Newborn	general search				
new-born	general search				
NMR	exact				
Neonat	general search				
**POSTNATAL INTERVENTIONS**					
postnatal - looking for postnatal care	general search				
PNC	exact				
breast	general search				
milk	general search				
**SMALL AND SICK NEWBORN**					
preterm	general search				
pre-term	general search				
prem*	general search				
Kangaroo	general search				
KMC	exact				
"birth weight" - looking for low birth weight	general search				
LBW	exact				
Sick and small newborn (small and sick *)	general search				
neonatal infection	general search				
"sepsis" - include only for newborn or neonatal sepsis	general search				
**QUALITY**					
quality	general				
Quality of care	exact				
Quality assurance	exact				
midw*	general				
referral	general				
"family" - looking for family-centred	general				

"respect" - looking for respectful care/disrespect&abuse	general				

GFF MNH analysis - template for counting mentions of mortality

Terms	How to search	# identified	Where is it mentioned (situation analysis, targets, M&E framework/indicat ors, other)	# identified	Where is it mentioned (situation analysis, targets, M&E framework/indicat ors, other)
**MATERNAL**					
maternal death	exact				
maternal mortality	exact				
MMR	exact				
**STILLBIRTH**					
stillbirth	exact				
SBR	exact				
perinatal death	exact				
fetal death	exact				
still birth	exact				
**NEWBORN**					
Newborn death	exact				
New-born death	exact				
Neonatal death	exact				
NMR	exact				
Neonatal mortality	exact				
Newborn mortality	exact				

Supplementary File 4. Detailed results

Table 4.1: Justification of scores by country by document

Country & document	Colour grading for the Ms	Description
**Burkina Faso**		
IC – pregnancy & childbirth	Mindset	Strongly included in the document. The high impact intervention package include improving the framework and conditions for the provision of basic and complementary EmONC (Emergency Obstetric and Neonatal Care) promoting maternal nutrition, improving the monitoring of mother-child relationships about HIV transmission, strengthening the monitoring of maternal deaths, strengthening the audit of maternal and neonatal deaths etc.
	Measures	Indicators mainly relate to maternal mortality and morbidity indicators. Ten indicators relate to maternal health are considered in the performance framework of the IC. These are: (i) maternal mortality ratio (deaths/100,000 live births); (ii) prevalence of anemia among women of childbearing age; (iii) skilled birth attendance rate; (iv) cesarean section rate; (v) mother-to-child transmission rate of HIV; vi) contraceptive prevalence among women of childbearing age; vii) contraceptive method use rate (%); viii) percentage of pregnant women attending ANC1 in the first trimester; ix) ANC4 use rate (%); x) proportion of audited maternal deaths.
	Money	The budget covers both maternal and newborn health. According to the so-called intermediate scenario, which is the one adopted, the cost of the "maternal and newborn health" program represents 24.5% of the total funding of the IC, which is the largest share of the budget (page 106). The total cost of the IC is estimated at XOF 1,036,451,660,029 (or approximately USD 1,884,457,564) over 5 years (2019-2023). A budget line has been defined for the "maternal and newborn health" program within this budget: XOF 254,089,903,556 (approximately S$461,981,643).
	Mindset	Mostly an integrated approach of the MNCH continuum. There are specific components of the project that include maternal health aspects, notably under sub-component on strengthening RMNCAH

PAD – pregnancy & childbirth		including EmONC and Primary Health Care (PHC). There is a focus on adolescent health linked to family planning due to the high rates of adolescent pregnancy.
	Measures	The results framework does not include mortality outcome indicators. There are two intervention coverage indicator relating to maternal health but both are relating to post-partum family planning services after delivery (one is specific to adolescent girls).
	Money	Under project component 2: Strengthening Delivery of Reproductive, Maternal, Newborn, Child and Adolescent Health and Nutrition (RMNCAH+N), there is US$35 million IDA, US$10 million GFF) with some targeted funds for maternal health including service delivery ($5m IDA, $8m GFF) which includes EmONC, PHC and postpartum family planning.
IC – postnatal and SSN care	Mindset	Newborn care is mentioned as not optimal. High-impact interventions to improve newborn health encompasses maternal and newborn care at home including the kangaroo method and breastfeeding promotion, monitoring the implementation of essential newborn care, scaling up maternal and newborn nutrition interventions, among others.
	Measures	Only one indicator related to newborns has been defined in the performance framework. It is the neonatal mortality rate.
	Money	The budget covers both maternal and newborn health. According to the so-called intermediate scenario, which is the one adopted, the cost of the "maternal and newborn health" program represents 24.5% of the total funding of the IC, which is the largest share of the budget (page 106). The total cost of the IC is estimated at XOF 1,036,451,660,029 (or approximately USD 1,884,457,564) over 5 years (2019-2023). A budget line has been defined for the "maternal and newborn health" program within this budget: XOF 254,089,903,556 (approximately S$461,981,643). Newborn care considered as part of “maternal” and not “child” health.
PAD – postnatal and SSN care	Mindset	Newborn care is assumed to be integrated into wider RMNCAH efforts under strengthening health services and is thorough document with some targeted areas e.g. EmONC and PHC and CRVS. Newborn care could be seen as part of community based program for IMCI and infant and young child feeding (linked to early initiation of breastfeeding), though it is not explicit.

	Measures	NMR not included as priority or specific indicators. There is only one indicator related to newborn in results framework for strengthening service delivery of RMNCAH+N: "% of newborns receiving birth certificate"
	Money	Under project component 2: Strengthening Delivery of Reproductive, Maternal, Newborn, Child and Adolescent Health and Nutrition (RMNCAH+N), there is US$35 million IDA, US$10 million GFF) with some targeted funds for newborn health including service delivery ($5m IDA, $8m GFF) which includes EmONC and PHC sub-components; as well as CRVS (US$9m IDA, $2m GFF).
**Cote d'Ivoire**		
IC – pregnancy & childbirth	Mindset	Maternal health, including care in pregnancy and childbirth, considered as entry point for health system strengthening and resource mobilization.
	Measures	Related indicators: ANC1 coverage in the first trimester of pregnancy, caesarean section rate, number of pregnant women tested for HIV, percentage of births attended by qualified staff, EmONC needs met, Number of maternal and neonatal deaths reported by health providers and community health workers (CHWs), ANC 4 coverage, maternal mortality ratio
	Money	no documentation identified
PAD – pregnancy & childbirth	Mindset	Intervention packages strongly mentioned with emphasis on EmONC, MPDSR and improving ANC access and quality. It mentions fetal heart rate monitoring. Nothing on dyad, mother-baby friendly, family centre or respectful care.
	Measures	Related Project Development Objective (PDO) indicator includes deliveries by skilled health personnel, ANC4, PNC mother
	Money	Component 2.2 Reproductive Health and Nutrition, which mentions newborn and stillbirth interventions, will receive US$14.7million.

IC – postnatal and SSN care	Mindset	Intervention packages have some mention, e.g. PNC, but are not well-described. Concepts mentioned: resuscitation, kangaroo care; breastfeeding. "low birth weight", "sick and small newborns" not mentioned
	Measures	Related indicators: neonatal mortality rate, under 6 months of exclusive breastfeeding, postnatal visits within 2 days.
	Money	no documentation identified
PAD – postnatal and SSN care	Mindset	Postnatal care is included, but only mentions for mother. No mention of SSN
	Measures	Related immediate results indicator includes postnatal care (CoPN but only mother mentioned)
	Money	Component 2.2 Reproductive Health and Nutrition, which mentions newborn and stillbirth interventions, will receive US$14.7million.
**Ethiopia**		
IC – pregnancy & childbirth	Mindset	Pregnancy and childbirth care are well described throughout the document as priorities. Baby and mother friendly or women friendly services are mentioned as well as content on respectful and compassionate maternity care. The focus on maternal health spans widely to include reproductive health (abortion and family planning), pregnancy care, childbirth care, and care following maternal adverse outcomes ie obstetric fistula.
	Measures	There are specific targets for MMR and related indicators e.g. ANC, SBA, c-sections, EMONC.
	Money	Budget provided by programme areas (Maternal, Newborn, Adolescent and Reproductive health, and later separated as MARH and Newborn and Child Health) and building blocks with different scenarios of budgets (base case, high investment). Maternal health receives 3.8% of proposed budget.
PAD – pregnancy & childbirth	Mindset	These interventions are included as key strategies (ANC, SBA, EmOC) or mentioned (abortion, PMTCT); no mention of mother-baby dyad or family-centered care.

	Measures	MMR is a key health indicator. DLI are included for maternal nutrition, ANC and delivery care with SBA.
	Money	Maternal nutrition, ANC, delivery care with SBA have allocations: $5million, $5 million (of $20m) and $10 million (of $64.43m), respectively.
IC – postnatal and SSN care	Mindset	Postnatal and newborn care described throughout the document as an integrated component of maternal and/or child health. There is a separate section on newborn in the background and most of the newborn-specific interventions included. Small and sick newborn not specifically included but interventions include for this population.
	Measures	There are specific targets for NMR and newborn specific indicators e.g. KMC, neonatal sepsis, resuscitation, IMNCI, PNC – as well as maternal linked indicators (SBA, c-sections, EMONC)
	Money	Budget provided by programme areas (Maternal, Newborn, Adolescent and Reproductive health, and later separated as MARH and Newborn and Child Health) and building blocks with different scenarios of budgets (base case, high investment). Newborn care is integrated into either child or maternal health. Newborn and child health receives 5.9% of base budget.
PAD – postnatal and SSN care	Mindset	The PAD is an update to a previous document with new components of focus, including PNC. No other specific newborn care intervention packages are described or included eg KMC, resuscitation
	Measures	PNC is included as a key strategy DLI. The indicator is to develop a PNC Directive to help increase coverage.
	Money	Allocation for PNC indicator is $5million
**Kenya**		
IC – pregnancy & childbirth	Mindset	Pregnancy and childbirth interventions are a core part of the document with strong focus on ANC and including of EmONC.
	Measures	Priority interventions subdivided in 6 areas: FP, MNH, malaria, HIV, immunization, child health. Results framework includes MMR, ANC, SBA.

	Money	Costed plan include related investments with MNH ~50% of proposed scale up with total KSH million 274546 for national (5 yrs)
PAD – pregnancy & childbirth	Mindset	Intervention packages well described in the document including mention of EmONC, midwifery and MPDSR.
	Measures	Core outcome measures: (b) Pregnant women attending at least four ANC visits (percentage); (c) Births attended by skilled health personnel (percentage)
	Money	Most financing to MNH through component 1 "Improving PHC Results (US$150m)" with specific elements of project targeted MNH (EmONC, midwifery, MPDSR, chlorhexidine)
IC – postnatal and SSN care	Mindset	Intervention packages described throughout document including specific newborn care (KMC, resuscitation, cord care, etc…)
	Measures	Results framework includes newborn care related indicators including targets for NMR and core coverage indicators for PNC, breastfeeding <6 months, birth registration/CRVS
	Money	Costed plan include related investments with MNH ~50% of proposed scale up with total KSH million 274546 for national (5 yrs)
PAD – postnatal and SSN care	Mindset	PNC mentioned once as part of key interventions. Project includes supporting the roll out of use of chlorhexidine for umbilical cord care but does not mention any other postnatal care or newborn care specific interventions.
	Measures	No related indicators
	Money	Most financing to MNH through component 1 "Improving PHC Results (US$150m)" with specific mention to small and sick newborn care, PNC and cord care.
**Liberia**		
	Mindset	Intervention packages are well integrated throughout document with focus on EmONC and MCH services, and links to adolescent health (due to high adolescent pregnancy rates). Key interventions all

IC – pregnancy & childbirth		mentioned and incorporated, as well as MNDSR. Family-oriented community based services included as a service delivery mode but no description. No mention of dyad or facility family centered care.
	Measures	Conceptual framework includes MMR as result. The results framework MMR, ANC4, SBA, Health facility delivery, PNC Mother, % hospitals with 100% CEmONC compliance, basic equipment availability (BEmONC).
	Money	Itemized cost per investment area includes items for maternal eg EmONC/MCH services US$34,5m; MNDSR $3m; also integrated into adolescent health; descriptions of community services include newborn components (ANC)
PAD – pregnancy & childbirth	Mindset	Intervention package is priority area described as part of the continuum. Focus on EmONC, MNDSR. Nothing clearly on mother-baby dyad; ANC identified as priority but no indicator.
	Measures	Results framework include indicators: Maternal death audits carried out routinely by PBF target hospitals (revised), Number of deliveries attended by skilled health personnel (millions) (new), quality index score - Quantity indicators for Hospital PBF include MNH related indicators: completed and assisted delivery, normal deliveries of at risk referrals, country referral slips returned to health facility
	Money	Mostly included under Component 1: Support to Quality Service Delivery Systems (US$13.7 million: 7.2 for QI in hospitals; 12.5 for rest).
IC – postnatal and SSN care	Mindset	PNC and newborn care is integrated throughout document, mostly via maternal, with focus on EmONC, CRVS, MNDSR and links to adolescent health and community services (linked to child). LBW and breastfeeding are mentioned as drivers of high mortality but not included as priority areas of investment with no mention of interventions for small and sick newborns.
	Measures	Conceptual framework includes NMR. The results framework includes NMR, PNC, birth registration. Breastfeeding not in the results framework.
	Money	Itemized cost per investment area includes items linked to newborn eg EmONC/MCH services US$34,5m; CRVS $3,5m; MNDSR $3M; descriptions of community services include newborn components (PNC)

PAD – postnatal and SSN care	Mindset	There is focus on EmONC, CRVS, MNDSR, PNC. Nothing on LBW, breastfeeding, small and sick newborn care.
	Measures	Results framework includes two new indicators: Neonatal death audits carried out routinely by PBF target hospitals; Newborns who have a postpartum contact with a health provider within 2 days of delivery in project target counties. Revised Health Facility Quality Index score improvement at Target PBF hospitals and facilities includes MNH related indicators: newborn referred for emergency newborn care treatment and treated.
	Money	Component 1: Support to Quality Service Delivery Systems (US$13.7 million: 7.2 for QI in hospitals; 12.5 for rest) includes intervention package. Component 4 with CRVS (US$1.8million with no break down only for CRVS).
**Malawi**		
IC – pregnancy & childbirth	Mindset	Maternal health has heavy focus in the background/context with some detail on ANC. Pregnancy is mentioned frequently but the focus of the IC and the costing is on human resources, drugs etc. rather than technical clinical interventions.
	Measures	The indicators are all organized by health system building block and are heavily focused on hardware / more general HR and systems upgrades (eg role clarity, supply chain and procurement optimization).<br><br>not specific interventions or time points in the continuum. Target for MMR.
	Money	Some of the costing linked to maternal health (construction of waiting homes, provision of TTV, big investment in hardware “Improve the availability of ANC and EMoNC equipment at facilities”.<br><br>Small funding for MDSR
PAD – pregnancy & childbirth	Mindset	Heavy focus on child health and U5 mortality / ECD and nutrition with little mention of care in pregnancy and time of birth.
	Measures	Pregnancy registration, Iron-folate supplementation for adolescent pregnancies, Reduction in rate of adolescent pregnancies
	Money	No related indicators

IC – postnatal and SSN care	Mindset	Mentioned in the background/context setting but focus is on the enabling environment.
	Measures	Using bottleneck analysis and identifying interventions to overcome by building block; not focused on key clinical interventions – measuring impact through u5 mortality reduction + stunting reduction
	Money	Some funding for CRVS/Birth registration
PAD – postnatal and SSN care	Mindset	Focus is on ECD with little mention of newborn care other than breastfeeding.
	Measures	One indicator – exclusive breastfeeding for 0-6 months.
	Money	Percentage increase on exclusive breastfeeding practices for children from 0-6 months ($1m)
**Mali**		
IC – pregnancy & childbirth	Mindset	Intervention package described as a priority area and throughout document, includes abortion, pregnancy care, childbirth care including mention of EmONC
	Measures	Comprehensive results framework with specific indicators for MNH including intervention packages in pregnancy and childbirth.
	Money	Budgeting organized by health system components with some focus on GBV and adolescents, nothing specific to MNH
PAD – pregnancy & childbirth	Mindset	Intervention package described as a priority area including mention of EmONC, MNDSR. Nothing clearly on mother-baby dyad; ANC identified as priority but no indicator.
	Measures	Indicators (coverage, health system and quality) includes ANC4, quality of care checklist, cause of death determined for maternal and child deaths.
	Money	Funding description focuses on access and quality of a broad package (RMNCH+N) and related PBF but nothing specific to MNH.
IC – postnatal and SSN care	Mindset	Postnatal care and newborn care included with focus on number of newborns receiving postnatal visit, newborn health, neonatal mortality rate, EmONC, neonatal septicemia, RMNAH+N, MNCH, NMR.
	Measures	Comprehensive results framework with specific indicators for MNH including PNC

	Money	Budgeting organized by health system components with some focus on GBV and adolescents, nothing specific to MNH<br><br>Itemized cost per investment area includes items linked to newborn eg EmONC/MCH services US$34,5m; CRVS $3,5m; MNDSR $3M; descriptions of community services include newborn components (PNC)
PAD – postnatal and SSN care	Mindset	Postnatal care mentioned once in background. Newborn care not specifically mentioned.
	Measures	No specific indicators
	Money	Funding description focuses on access and quality of a broad package (RMNCH+N) and related PBF but nothing specific to MNH.
**Nigeria**		
IC – pregnancy & childbirth	Mindset	Intervention package described as a priority area and throughout document, includes comprehensive list of interventions for each package. One mention around disrespect and abuse during childbirth.
	Measures	Results framework is more limited to a select number of indicators, which include monitoring number of births and proportion of skilled birth attendants.
	Money	MNCH included as priority investment area – committing to pay for all MNH in rural areas. Funds available to MNCH USD200.98 million. No specific intervention packages described.
PAD NSHIP – pregnancy & childbirth	Mindset	Intervention care packages mostly integrated broadly as maternal and child health. Some focus on care at birth and mentions around pregnancy care.
	Measures	Skilled birth attendance is a core project indicator. One intermediate indicator includes number of pregnant women tested for HIV during antenatal care
	Money	Funding for general health service strengthening with sections on paediatric and obstetric care (amounts not specified)

PAD Nutrition – pregnancy & childbirth	Mindset	Pregnancy related interventions mentioned in relation to adolescence or focused on nutrition sensitive antenatal care. Fistula mentioned in relation to adolescent pregnancy.
	Measures	Indicators related to intervention packages include: Pregnant women who consume a minimum of 90 iron-folic acid tablets (proportion) and Pregnant women who receive intermittent presumptive treatment for malaria [at least three doses] (proportion)
	Money	Funding description includes antenatal care.
PAD HUWE – pregnancy & childbirth	Mindset	The Fee For Service component of the project includes four maternal health interventions for pregnant women (antenatal care, labor and delivery, emergency obstetric and neonatal care, and caesarean section). These are included as part of Component 1 of the project (Strengthening Primary Health Care)
	Measures	The project indicators focus on the Fee For Service Mechanism which includes the four maternal health interventions.
	Money	Funding for Component 1 includes specifically description of these intervention packages with USD$8.9 million allocated.
IC – postnatal and SSN care	Mindset	MNH included throughout including postnatal care and SSN, mostly integrated as part of basic package of interventions and includes comprehensive list of interventions for each package. Burden of preterm birth described. SSN care interventions included as interventions (KMC, sepsis, resuscitation) as key intervention.
	Measures	Results framework is more limited to a select number of indicators and none are specific to PNC or SSN
	Money	MNCH included as priority investment area – committing to pay for all MNH in rural areas. Funds available to MNCH USD200.98 million. No specific intervention packages described.
PAD NSHIP – postnatal and SSN care	Mindset	Postnatal care and SSN not mentioned.
	Measures	Postnatal care and SSN not mentioned.
	Money	Postnatal care and SSN not mentioned.
	Mindset	Intervention package mostly focused related to nutrition and linked to breastfeeding.

PAD Nutrition – postnatal and SSN care	Measures	Related indicator includes exclusive breastfeeding from 0-6 months
	Money	Description of funding includes counselling related to breastfeeding.
PAD Huwe – postnatal and SSN care	Mindset	Intervention packages mentioned only in relation to maternal and child health with only one mention of PNC and no inclusion of SSN care.
	Measures	There are no specific indicators.
	Money	These intervention packages are described in a footnote related to the predefined benefit package of high impact maternal and health services but not specifically described.
**Senegal**		
IC – pregnancy & childbirth	Mindset	Strong inclusion of intervention packages in document.
	Measures	Results framework includes MMR, % of births attended by skilled personnel, contraceptive prevalence rate (% of women aged 15-49 in union currently using a modern contraceptive method).
	Money	Budget allocated specifically to the maternal health sub-priority is 2,185,846,791 FCFA (about USD 4,163,518) out of 496,132,650,133 FCFA (about USD 945,014,572). The budgets allocated to maternal and neonatal health activities were combined in some cases.
PAD – pregnancy & childbirth	Mindset	Intervention packages mostly integrated as part of the broader RMNCAH+N approach. Some specifics re care at birth & adolescent pregnancy. Nothing on abortion, PMTCT.
	Measures	Includes core indicator - Percentage of pregnant women having 4 antenatal care visits at standard quality (percentage) as well as related intermediate indicators<br><br>Number of deliveries<br><br>% deliveries in high capacity health facilities<br><br>% births in health centers with functional EmONC base

	Money	Packages described under "improving availability of RMNCAH-N services of adequate quality – supporting HR and strengthening clinical competency and capacity to deliver quality of care. Not able to dissagregate between MNH (total $60m; $55m IDA; $5m GFF)
IC – postnatal and SSN care	Mindset	Specific interventions included as part of situation analysis eg PNC, resuscitation, LWB. SSN not mentioned.
	Measures	Specific indicators include neonatal mortality rate, number of children born to HIV-positive mothers put on ART prophylaxis, % of newborns who received the immediate care package at birth, proportion of fresh stillbirths, proportion of infants breastfed from the first hour of birth, coverage rate of PNC)
	Money	The budget of the IC is estimated at 496,132,650,133 FCFA (about USD 945,014,572) and the "Neonatal Health" part has a budget of 10,903,063,542 FCFA (USD 20,767,740). There are also budget lines common to mothers and newborns, and budget lines common to all RMNCAH targets.
PAD – postnatal and SSN care	Mindset	Intervention packages mostly integrated as part of the broader MNCAH approach. Specific interventions and indicators to improve newborns health are found, notably immediate newborn care, EmONC, and postanatal care. Breastfeeding not mentioned. LBW mentioned once linked to AH). Sick child care/newborn care management included but not specific to preterm or SGA.
	Measures	Intermediate indicator on immediate newborn care package
	Money	Packages described under "improving availability of RMNCAH-N services of adequate quality – supporting HR and strengthening clinical competency and capacity to deliver quality of care. Not able to dissagregate between MNH (total $60m; $55m IDA; $5m GFF)
**Tanzania**		
IC – pregnancy & childbirth	Mindset	Strong inclusion throughout document with large focus on EmONC, MPDSR, ANC and other related interventions.
	Measures	Comprehensive list of targets and indicators (mortality, coverage along continuum) including ANC and Skilled Birth Attendance as well as many other related indicators.
	Money	Costing plan comprehensively include intervention packages.

PAD – pregnancy & childbirth	Mindset	MNCH as a thematic area is well present in the document across areas. The intervention packages are well described in background and lives saved analyses and incorporated into activities as part of broader PHC approaches.
	Measures	Specific projected linked indicators include ANC4, IPTp, institutional deliveries
	Money	The service delivery indicators (primarily wrapped into DLI 3 and 4) are only 2.3% of funding dedicated. RBF as the core mechanism for achieving impact in MNH (including through scorecards)
IC – postnatal and SSN care	Mindset	Intervention packages well described in the document including sections and interventions for small and sick newborn care.
	Measures	Comprehensive list of targets and indicators (mortality, coverage along continuum) including PNC and SSN care (eg KMC).
	Money	Costing plan comprehensively include intervention packages.
PAD – postnatal and SSN care	Mindset	MNCH as a thematic area is well present in the document across areas. The intervention packages are well described in background and lives saved analyses and incorporated into activities as part of broader PHC approaches.
	Measures	No specific indicators for these packages.
	Money	The service delivery indicators (primarily wrapped into DLI 3 and 4) are only 2.3% of funding dedicated. RBF as the core mechanism for achieving impact in MNH (including through scorecards)
**Uganda**		
IC – pregnancy & childbirth	Mindset	Intervention packages are a core part of the document with strong focus on ANC and including of EmONC.
	Measures	MMR included as a goal. Maternal related coverage indicators included (ANC, PMTCT, IPT, ITN, institutional deliveries, PNC mother).

	Money	The costing analysis includes MNH interventions via "packages". Description of packages include MNH elements e.g. maternity wings, midwives, B and CEmOC. Annex includes some break down of service packages including SBA, post abortion and sepsis management.
PAD – pregnancy & childbirth	Mindset	Intervention packages are core to the RMNCAH strategy.
	Measures	Project development objective indicators include births delivered by skilled health personnel and pregnant women who received IPT2. Reported maternal deaths that are audited also included.
	Money	Description of project components and linked funds include maternal but there is not a standalone focus on these intervention packages.
IC – postnatal and SSN care	Mindset	Intervention packages are a core part of the document including PNC and newborn care
	Measures	NMR included as a specific goal. Other newborn related coverage indicators included (PNC, early initiation of breastfeeding, management of severe neonatal infections, KMC).
	Money	The costing analysis includes MNH interventions via "packages" but nothing specific to newborn or postnatal care.
PAD – postnatal and SSN care	Mindset	Intervention packages are included as core to RMNCAH strategy though they are less mentioned throughout compared to pregnancy and childbirth packages.
	Measures	No specific indicators for these packages.
	Money	Description of project components and linked funds include maternal but there is not a standalone focus on these intervention packages.

Key:
ANC - Antenatal Care
BEMONC – Basic Emergency Obstetric and Neonatal Care
CEMONC - Comprehensive Emergency Obstetric and Neonatal Care
DLI – Disbursement Linked Indicators
EMOC - Emergency Obstetric Care
EMONC - Emergency Obstetric and Neonatal Care
IPT - intermittent preventive treatment

ITN - Insecticide-treated bed nets
KMC – Kangaroo Mother Care
LBW – low birthweight
MMR – Maternal Mortality Ratio
MPDSR – maternal and perinatal death surveillance and response
NMR – Neonatal Mortality Rate
PDO – Project Development Objectives
PHC – primary health care
PNC - Postnatal Care
RBF – results based financing
RMNCAH+N - Reproductive Maternal Newborn Child Adolescent Health plus Nutrition (sometime only some components included eg MNCH, MNH, RMNCH)
SBA – skilled birth attendant
SGA – small for gestational age
SSN – small and sick newborn

Table 4.2: Results for mentions of interventions packages in the MNH continuum of care

Continuum of care category	Total mentions in ICs (% total counts)	Total mentions in PADs (% total counts)
Pregnancy interventions	1350 (47%)	447 (43%)
Childbirth care interventions	1023 (35%)	462 (42%)
Postnatal interventions	305 (11%)	124 (11%)
Small and sick newborn interventions	222 (8%)	44 (4%)

Table 4.3: Summary results by country, by document, by framework component

	Summary	Mindset	Measures	Money
Burkina Faso	Consistent<br><br>Consistent framing around MNH with both documents integrating terms throughout.<br><br>IC: comprehensive including specific mortality targets (for maternal and newborn) and coverage targets and allocated budget for MNH<br><br>PAD: holistic approach focusing on a broad range of RMNCAH issues through health system strengthening approaches, notably program based financing and free-healthcare policies.	Consistent<br><br>IC: MNH described throughout document mostly as integrated. No mention of stillbirths beyond related interventions (EmONC and audit).<br><br>Targets include maternal and newborn mortality.<br><br>PAD: MNH mentioned as mostly integrated (MNCH) Areas of focus include EmONC, family planning especially for adolescents, PHC, CRVS, and ECD.	Not consistent<br><br>IC: Mostly maternal indicators included; NMR included; nothing specific to stillbirth.<br><br>PAD: No mortality outcome indicators, some maternal health indicators; one newborn related indicator (birth certificate)<br><br>Types of indicators<br>IC: Results framework – mortality and coverage<br>PAD: Project Development Objective indicators (numbers and coverage)	Consistent<br><br>Consistent in terms of proportion allocation for MNH: 24.5% of the total funding budgeted in IC for MNH; 30% allocated in the PAD.<br><br>IC: the budget for MNH are put together estimated at USD 461,981,643 (5 years)<br><br>PAD: MNH included in one component: Strengthening Delivery of RMNCAH+N (US$35 million IDA, US$10 million GFF)
Cote d'Ivoire	Consistent framing	Consistent framing	Not consistent	Not consistent

	IC focuses more on governance and overall health system strengthening, which aligns with the PAD priorities. MNH is included in mindset within both documents with more focus on pregnancy and childbirth interventions.	IC: MNC considered as entry point for health system strengthening and resource mobilization.<br><br>Targets include maternal mortality. Neonatal mortality included as indicator.<br><br>PAD: MH is a main focus and mentioned throughout. Newborn mentioned mostly linked to maternal. There is strong emphasis on EmONC, resus, MPDSR, PNC and improving ANC access and quality. Pregnancy diagnostics linked to SB.	IC: Comprehensive results framework with mortality and coverage indicators<br><br>PAD: Related project indicators includes deliveries by skilled health personnel, ANC4, PNC mother<br><br>Types of indicators<br>IC: n/a<br>PAD: Project Development Objective indicators (numbers and coverage)	IC: No budgetary details regarding MNH specifically.<br><br>PAD: Component 2.2 Reproductive Health and Nutrition, which mentions newborn and stillbirth interventions, will receive US$14.7million from IDA (7% of total PAD)
Ethiopia	Partly consistent<br><br>Ethiopia documents both include MNH with related targets and indicators as well as budgets/allocations to related interventions. Documents not fully aligned.<br><br>IC: broad and comprehensive addressing issues across the continuum of care, including care for small and sick newborns, with an emphasis on quality improvement.<br><br>PAD: narrower focus as it is readjusting a previous proposal for RMNCAH. It includes some measures and money for MNH but no mention of small and sick newborn issues/programs and stillbirth/perinatal, which were included in the IC.	Partly consistent<br><br>IC: MNH described throughout with more focus on maternal. No mention of stillbirth beyond target. Mostly integrated in approach to MNH but more attention to maternal. No mention of dyad but includes respectful care and mother/baby friendly services and respectful maternity care.<br><br>Targets include maternal and newborn mortality.<br><br>PAD: MNH included but mostly integrated (MNH). Focus on PNC	Partly consistent<br><br>IC: Comprehensive set of MNH indicators included, including SB<br><br>PAD: MMR key indicator; **DLI** for maternal nutrition, ANC, SBA and PNC<br><br>Types of indicators<br>IC: mortality, coverage, process indicators<br>PAD: Disbursement Linked Indicators (coverage and process indicators)	Partly consistent<br><br>IC: Costing includes MNCH as program areas with 3.8% for maternal related and 5.8% for newborn related of base cost scenario USD 15,6 million (5 yrs)<br><br>PAD: Allocation for related DLI Maternal nutrition, ANC, delivery care with SBA and PNC for newborn (USD 25 million total) 17% of the total funding budgeted relate to MNH

		and includes KMC &resus. No mention of stillbirth, FCC or dyad.		
Kenya	Partly consistent<br><br>IC: broad with strong MNH component throughout (eg less on newborn specifically); very focused on technical/clinical aspects and indicators in each period – limited integration across the continuum or consideration of patient experience.<br><br>PAD: focus on PHC and quality/regulation. Less focus on MNH with little on newborn.<br><br>Quality is mentioned frequently in both docs and viewed as important	Partly consistent<br><br>IC: MNH described throughout as integrated but with more focus on maternal. Clear focus on quality and HSS with some priority to EmONC and mention of need for newborn nurseries in level 4 hospitals. Stillbirth incorporated. Limited mention of CoC, referral, respectful, FCC or dyad.<br><br>Targets include maternal and newborn mortality.<br><br>PAD: MNH included but more emphasis on maternal (newborn & stillbirth mentioned as part of burden); Midwifery included but limited mention of key interventions; no mention FCC or dyad.	Partly consistent<br><br>IC: Results framework includes MNH related indicators including targets for MMR and NMR and core coverage indicators ANC, SBA, PNC, breastfeeding.<br><br>PAD: PDO Level Results Indicators include ANC, SBA and immunization; no outcome indicator for newborn health<br><br>Types of indicators<br>IC: Results framework (mortality, coverage, other)<br>PAD: Project Development Objective indicators (5 indicators only – all health coverage)	Partly consistent<br><br>IC: Costed plan MNH related HSS investments with MNH ~50% of proposed scale up with total KSH million 274546 for national (5 yrs)<br><br>PAD: Most financing to MNH through component 1 "Improving PHC Results (US$150m) with specific elements of project targeted MNH (EmONC, midwifery, MPDSR, chlorhexidine)
Liberia	Consistent<br><br>MNH integrated and content aligned. Missing some core elements needed to address MNH notably small and sick newborn care and respectful care.<br><br>PAD is a revision on previous one.<br><br>Core MNH indicators are included, though ANC absent in PAD.	Consistent<br><br>IC: Holistic strategy with MNH integrated throughout<br><br>Targets include maternal and newborn mortality.<br><br>PAD: MNH mentioned throughout mostly integrated. Focus on EmONC, CRVS,	Partly consistent<br><br>IC: Results framework includes MMR, ANC4, SBA, Health facility delivery, PNC Mother, % hospitals with 100% CEmONC compliance, basic equipment availability (BEmONC), NMR, PNC, birth registration but not stillbirth	Consistent<br><br>IC: MNH investment integrated into wider service delivery. Total national cost of plan (5 yrs) USD298.7 million. Phase One of IC for RMNHC (focus on 6 counties) is USD79.8 million – 47% of which

	MNH specific investments are integrated into broader quality service delivery system efforts.	MNDSR, PNC. Nothing on SSN, dyad or FCC. Stillbirth mentioned once as part of burden.	PAD: PDO outcome indicators – long list including MNH indicators (e.g. audit, skilled deliveries, PNC newborn), Hospital PBF include MNH related indicators<br><br>Types of indicators<br>IC: Results framework (mortality, coverage, other)<br>PAD: Project Development Objective indicators (outcome, coverage, health systems)	itemized to EmONC, MNH and MPDSR.<br><br>PAD: Most financing to MNH through component 1 "Support to Quality Service Delivery Systems ((US$13.7 million in new PAD).<br><br>MNH integrated - 86%
Malawi	Partly consistent<br><br>IC: focus is more broadly on health system building blocks and enabling environment/health systems strengthening; less attention to specific topics such as MNH.<br>PAD: focus on ECD with little mention of MNH beyond breastfeeding.<br><br>*Note: IC written after the PAD*	Partly consistent<br><br>IC: Strong focus on maternal health with less focus on newborn health.<br><br>Targets include maternal and newborn mortality.<br><br>PAD: focus on ECD with little mention of newborn other than breastfeeding	Partly consistent<br><br>IC: Outcome indicators aligned indicators to their Health Sector Strategy (5 years), eg MMR, NMR, IMC, U5MR, Ferity, Underweight<br><br>PAD: Results framework with PDO indicators and some DLI indicators – few linked to MNH ie breastfeeding, adolescent pregnancy<br><br>Types of indicators<br>IC: Mortality<br>PAD: Project Development Objective indicators & DLI (coverage, health systems)	No consistent<br><br>IC: Costing done through building blocks with some big investments linked to MNH eg equipment for EmONC. Small funding for MDSR.<br>USD 928 million (3yrs)<br><br>PAD: No DLI for maternal. US$1 million DLI for exclusive breastfeeding 6 months
Mali	Partly consistent<br><br>IC: MNH embedded strongly with specific sections and indicators.	Not consistent	Partly consistent<br><br>IC: Comprehensive results framework with specific	Consistent<br><br>IC: Budgeting organized by health system

	PAD: MNH included as broader continuum only other than one indicator (ANC4)	IC: MNH included throughout document with some specific sections for each.<br><br>Targets include maternal and newborn mortality.<br><br>PAD: MNH included as part of broader RMNCAH+N package. Nothing specific other than ANC4 being listed as an indictor.	indicators for MNH including small and sick newborn indicators<br><br>PAD: PDO indicators (coverage, health system and quality) includes ANC4, quality of care checklist, cause of death determined for maternal and child deaths.<br><br>Types of indicators<br>IC: Mortality, coverage, health system<br>PAD: Project Development Objective indicators(coverage, health systems)	components with some focus on GBV and adolescents, nothing specific to MNH<br>Total budget for IC: USD 969000<br><br>PAD: Funding description focuses on access and quality of a broad package (RMNCH+N) and related PBF but nothing specific to MNH.
Nigeria*	Consistent<br><br>IC – focus is on health systems with few mentions of MNH as part of broader RMNCAH basic package<br><br>3 PADs include GFF funding.<br>• NSHIP PAD focuses on one conflict-affected region (NE – 5 States) and health system strengthening;<br>• Nutrition PAD focuses on stunting in 12 states with some interventions for maternal care (folic acid and iPTP) and breastfeeding;<br>• HUWE PAD focused on 3 States basic health provision fund with	Partly consistent<br><br>IC: MNH included throughout; mostly integrated as part of basic package of interventions. Separate sections on maternal and newborn in background. One mention stillbirth. SSN included.<br><br>Targets include maternal and newborn mortality.<br><br>PAD NSHIP: MNH mostly integrated as "Maternal and child" with SBA used as indicator/measure. Focus is on PHC.	Partly consistent<br><br>IC: Results framework with target indicators (including SBA and quality index)<br><br>PAD: PDO indicators includes SBA and QoC measures; revised PDO indicators mostly broader health systems/structural<br><br>Types of indicators<br>IC: Results framework 7 indicators (coverage and indices)<br><br>PAD NSHIP: 5 PDO indicators (with changes to	Partly consistent<br><br>IC: MNCH included as priority investment area – committing to pay for all MNH in rural areas. Funds available to MNCH USD200.98 million (from GFF, Government, BMGF, TBD) (years not specified)<br><br>PAD: Funding for general health service strengthening with sections on paediatric and obstetric care.<br><br>PAD NSHIP: Funding for general health service

	priority on care in pregnancy and aa birth<br><br>Nigeria's IC includes MNH with clear attention to maternal health and some inclusion of newborn health interventions and mentions. The 3 PADs range in focus (nutrition, primary health care, and basic health provisions including MNH services – mostly care in pregnancy and at birth).<br><br>*Note: PAD NSHIP is revised WB document providing additional credit and revisions to content and indicators.*	PAD Nutrition: MNH interventions integrated as part of broader strategy for addressing stunting<br><br>PAD HUWE: MNH care in pregnancy and at birth are prioritized throughout.	measurement in revised PAD)<br><br>PAD Nutrition: 11 PDO indicators (3 for MNH) and 42 DLIs<br><br>PAD HUWE: PDO indicators linked to health systems and finance mechanisms	strengthening with sections on paediatric and obstetric care (amounts not specified)<br><br>PAD Nutrition: Project components include MNH linked to nutrition (USD180.15m million) 47%<br><br>PAD HUWE: Project component includes fee-for-service approach linked to quantity of services – including MNH (USD$8.9 million) 45%
Senegal	Partly consistent<br><br>Broad health system approach with some links to MNH interventions as well as other areas along continuum, esp adolescent health.<br><br>IC: MNH specifically mentioned with some aspects absent (SSN, FCC)<br><br>PAD: MNH mostly integrated and focus on adolescent pregnancy	Consistent<br><br>IC: MNH mentioned throughout with focus on quality of care overall.<br><br>Targets include maternal and newborn mortality.<br><br>PAD: MNH mostly integrated as part of the broader RMNCAH+N approach. Some specifics re care at birth & adolescent pregnancy. Nothing on FCC, SSN, abortion, PMTCT.	Partly consistent<br><br>IC: Results framework includes MMR, NMR, morbidity and coverage indicators (ANC, SBA) and ANC quality score. Related health system indicators (eg access to EmONC)<br><br>PAD: Project Development Objective indicators includes quality index, ANC, newborn package, EmONC<br><br>Types of indicators<br>IC: Results framework (mortality, coverage, other)<br>PAD: Project Development Objective indicators (5 core	Partly consistent<br><br>IC: MNH included in budget allocation with 2.64% (USD 24.9 million) though other sections may also cover MNH related costs.<br><br>PAD: Broader focus for investment in health system. Allocation for MNH integrated in multiple components. Component 1 – improving services of adequate quality includes explicitly (USD $60 million); Component 2 – adolescent health (USD 25

			coverage; 4 intermediate indicators)	million); reforms (USD 65 million)
Tanzania	Not consistent<br><br>IC: strong technical document focusing on key MNH interventions with large focus on EmONC with linked indicators and budget allocations<br>PAD: Focus on primary health care and broad system strengthening over higher-level MNH technical content.<br><br><br>*Note: PAD precedes the IC by a year*	Partly consistent<br><br>IC: MNH incorporated throughout document with large focus on EmONC.<br><br>Targets include maternal and newborn mortality.<br><br>PAD: MNH integrated throughout with broader focus on PHC.	Partly consistent<br><br>IC: Comprehensive list of targets and indicators (mortality, coverage along continuum)<br><br>PAD: 7 DLI indicators – MNH specifically included in 2 – focus on PHC more broadly<br><br>Types of indicators<br>IC: Mortality, coverage, health systems<br>PAD: DLIs (performance, capacity building)	Not consistent<br><br>IC: MNH activities largest area of funding as a whole with detailed costing by specific sub-interventions. Newborn also in the child health budget line. 5 year plan<br><br>PAD: MNH service delivery indicators primarily wrapped into DLI 3 and 4 (182m of 300m) – only 2.3% of funding dedicated to MNCH<br><br>RBF as the core mechanism for achieving impact in MNH (including through scorecards)
Uganda	Consistent<br><br>Documents related and take a holistic approach to addressing RMNCAH on improving service delivery and health system aspects, with specific attention to human resources (specifically midwives) and scaling up results based financing mechanisms, and improving quality of care. More mention of maternal elements than newborn related components.	Consistent<br><br>IC: MNH described throughout the documents. Mostly integrated with more attention to maternal. No mention of FCC, respectful care, dyad.<br><br>Targets include maternal and newborn mortality.	Partly consistent<br><br>IC: Results framework includes multiple MNH related indicators. Goals/targets include MMR, NMR and stillbirth rate.<br><br>PAD: Multiple maternal health related indicators included in project	Partly consistent<br><br>IC: Costing done by level of care with MNH elements describes (maternal wing, midwives, EmONC). Total USD 1,918 million

	IC: MNH is a strong component but priority weakens, especially for newborn/stillbirth, as it moves to the investment.<br><br>PAD: MNH throughout with more priority given to maternal health.	PAD: MNH described throughout. Little on small and sick newborn care. No mention of FCC, respectful care, dyad.	development indicators (ANC, SBA, maternal deaths audited, c-sections)<br><br>Types of indicators:<br>IC: Results framework (mortality, coverage, other)<br>PAD: Project Development Objective indicators (6 indicators)	PAD: Project components include MNH including PHC (USD 43million), System strengthening (USD 54.5 million), and capacity strengthening (USD 5 million)

Table 4.4: Frequency of mentions

a) Frequency of mortality outcomes mentioned

		Investment case				PAD		
Countries and territories	IC Date	Maternal	Newborn	Stillbirth	PAD Date	Maternal	Newborn	Stillbirth
Burkina Faso	Jun-19	53	52	0	Jul-18	4	2	0
Côte d'Ivoire	Apr-19	26	13	1	Mar-19	25	1	3
Ethiopia	Oct-15	28	8	1	Apr-17	15	4	0
Kenya	Jan-16	31	13	13	May-16	30	8	1
Liberia	No date	23	39	3	Jan-17	20	22	1
Malawi	Nov-19	19	10	1	Nov-18	2	1	1
Mali	2019	81	43	1	Feb-19	3	1	0
Nigeria - NSHIP	No date	16	2	2	May-16	2	0	0
Nigeria - Nutrition					May-18	4	0	0

Nigeria - HUWE					Aug-18	4	1	0
Senegal	Jun-19	158	45	1	Sep-19	13	9	1
Uganda	Apr-16	48	34	16	Jul-16	32	7	6
United Republic of Tanzania	Jun-16	45	48	36	May-15	38	27	2

b) Frequency of search terms mentioned

Investment case

**Terms**	**How to search**	**Burkina Faso**	Cote d'Ivoire	Ethiopia	Kenya	Liberia	Malawi	Mali	Nigeria	Senegal	Tanzania	Uganda	TOTAL
**MATERNAL**													
maternal	general	89	56	65	99	103	39	157	45	196	249	216	1314
MMR	exact	12	7	6	3	0	0	16	3	7	22	12	88
mother		71	17	**25**	27	31	5	50	14	60	52	40	392
**PREGNANCY INTERVENTIONS**			0										0
antenatal	general	23	13	6	8	30	4	12	8	15	23	19	161
ANC	exact	29	4	16	18	26	22	9	9	28	278	15	454
PMTCT	exact	13	0	9	4	5	2	9	7	5	17	9	80

abortion	general	30	2	9	9	39	4	12	1	11	8	24	149
pregnan* (pregnant; pregnancy)	general	53	15	**24**	41	67	35	46	17	38	90	80	506
**STILLBIRTH**			0										0
Stillb*	general search	0	0	0	11	2	0	1	1	1	10	9	35
fetal	general search	1	0	0	2	0	3	0	0	0	0	0	6
still birth	general search	3	0	1	2	0	0	0	0	0	3	0	9
**CHILDBIRTH CARE**			0										0
skilled birth attend	general	8	7	16	7	10	0	16	8	32	7	10	121
SBA	exact	0	0	4	10	5	0	0	0	17	8	7	51
skilled attend	general	0	0	2	4	0	0	0	1		6		13
"delivery"~	general	32	16	75	97	10	0	41	8	42	67	15	403
EmOC	general	42	3	1	0	0	0	1	0	14	3	1	65
EmONC	general	2	2	8	24	53	0	5	4	2	26	10	136
EmNOC	general	0	0	0	0	0	6	0	0	1	0	0	7
obstetric	general	19	9	15	13	26	12	10	5	10	28	11	158
resus	general search	2	2	1	5	0	0	8	2	2	14	7	43
Perinatal	general search	0	2	1	0	1	0	1	1	0	12	8	26

**NEWBORN**			0										0
Newborn	general search	31	7	21	53	2	13	42	17	30	297	69	582
new-born	general search	16	0	1	1	52	0	0	9		1	4	84
NMR	exact	14	10	2	4	0	0	17	0	9	14	8	78
Neonat	general search	56	35	28	34	60	23	141	17	72	32	46	544
**POSTNATAL INTERVENTIONS**			0									8	8
postnatal - looking for postnatal care	general search	8	6	6	8	21	2	1	6	4	17	8	87
PNC	exact	9	0	7	7	6	7	1	3	1	12	4	57
breast	general search	37	3	2	14	11	5	5	2	7	56	11	153
milk	general search	1	1	0	1	0	0	0	0	1	4	0	8
**SMALL AND SICK NEWBORN**			0										0
preterm	general search	8	1	0	6	2	0	8	1	5	12	7	50
pre-term	general search	0	0	0	0	0	0	0	1		0	0	1
prem*	general search	2	0	2	3	4	2	0	3		0	5	21

Kangaroo	general search	7	1	1	5	0	0	3	1	2	9	5	34
KMC	exact	3	0	2	0	0	0	0	0	2	15	4	26
"birth weight" - looking for low birth weight	general search	11	0	1	1	2	0	6	0	1	8	2	32
LBW	exact	4	0	0	0	0	0	0	0		4	4	12
Sick and small newborn (small and sick *)	general search	2	0	0	0	0	0	0	0	1	2	0	5
neonatal infection	general search	0	0	0	4	0	0	2	0	3	0	3	12
"sepsis" - include only for newborn or neonatal sepsis	general search	1	1	3	5	3	3	0	4	2	3	4	29
**QUALITY**			0										0
quality	general	92	49	306	101	82	84	85	28	103	71	66	106<br>7
Quality of care	exact	12	10	15	4	13	2	6	9	11	4	10	96
Quality assurance	exact	0	7	21	8	6	3	0	0	0	0	2	47
midw*	general	2	7	4	11	23	2	2	1	27	0	22	101
referral	general	7	2	16	10	21	5	4	4	7	26	16	118
"family" - looking for family-centred	general	0	1	0	0	0	0	0	0	0	0	0	1
"respect" - looking for respectful care/disrespect&abuse	general	3	0	26	8	1	0	0	1	6	1	1	47

Project appraisal documents

**Terms**	**How to search**	**Burkina Faso**	Cote d'Ivoire	Ethiopia	Kenya	Liberia	Malawi	Mali	PAD - NSHIP	PAD - HUWE	PAD - Nutrition	Senegal	Tanzania	Uganda	TOTAL
**MATERNAL**															
maternal	general	31	70	84	66	49	23	22	18	26	17	159	158	70	793
MMR	exact	0	3	2	0	3	0	0	0	0	0	3	6	0	17
mother		8	12	5	14	4	10	1	4	1	30	17	7	**25**	138
**PREGNANCY INTERVENTIONS**															0
antenatal	general	4	12	24	3	7	2	8	2	6	9	13	9	6	105
ANC	exact	6	16	2	14	12	11	1	2	0	0	0	18	3	85
PMTCT	exact	2	0	0	3	1	10	0	0	0	0	0	3	1	20
abortion	general	1	2	4	2	7	0	0	0	0	0	3	0	5	24
pregnan* (pregnant; pregnancy)	general	13	26	35	26	11	40	10	9	1	59	36	14	**26**	306
**STILLBIRTH**															0
Stillb*	general search	0	1	0	1	1	1	0	0	0	0	0	0	0	4
fetal	general search	0	1	0	0	0	0	0	0	0	0	0	0	0	1
still birth	general search	0	0	0	0	0	0	0	0	0	0	0	0	0	0
**CHILDBIRTH CARE**															0
skilled birth attend	general	1	7	30	6	6	0	0	6	9	0	2	3	4	74
SBA	exact	0	0	4	1	0	0	0	0	0	0	0	0	0	5
skilled attend	general	0	0		0	0	0	2		0	0	0	2	0	4

"delivery"~	general	0	15	9	67	2	0	0	3	0	0	3	118	5	222
EmOC	general	0	0	0	0	0	0	0	0	0	0	0	0	0	0
EmONC	general	6	8	0	0	11	0	0	0	0	0	8	1	7	41
EmNOC	general	0	0	0	0	0	0	0	0	0	0	0	0	0	0
obstetric	general	6	18	12	5	15	0	2	4	3	2	5	8	10	90
resus	general search	0	6	0	0	0	0	0	0	0	0	0	0	0	6
Perinatal	general search	1	4	0	2	1	0	1	0		0	3	2	6	20
**NEWBORN**															0
Newborn	general search	17	15	8	23	19	3	2	1	8	0	9	7	6	118
new-born	general search	0	0	0	0	1	0	0	0		0	0	0	2	3
NMR	exact	0	0	0	0	0	0	0	0		0	0	1	2	3
Neonat	general search	6	24	11	17	33	3	12	2	5	5	19	95	23	255
**POSTNATAL INTERVENTIONS**															0
postnatal - looking for postnatal care	general search	5	9	25	2	7	1	1	0	1	0	5	2	3	61
PNC	exact	2	0	0	4	3	0	0	0	0	0	0	0	3	12
breast	general search	5	1	0	1	1	20	0	0	0	19	0	0	1	48
milk	general search	0	1	0	0	0	0	0	0	0	2	0	0	0	3
**SMALL AND SICK NEWBORN**															0
preterm	general search	0	0	0	0	0	1	0	0	0	0	0	2	0	3
pre-term	general search	0	0	0	0	1	0	0	0	0	0	0	0	0	1

prem*	general search	0	3	0	0	4	0	0	0	0	0	0	0	2	9
Kangaroo	general search	0	0	0	0	0	0	0	0	0	0	0	0	5	5
KMC	exact	0	0	0	0	0	0	0	0	0	0	0	0	4	4
"birth weight" - looking for low birth weight	general search	0	0	0	1	1	2	1	0	0	1	1	1	1	9
LBW	exact	0	0	0	0	0	0	0	0	0	0	0	0	1	1
Sick and small newborn (small and sick *)	general search	0	0	0	1	0	0	0	0	0	0	0	0	0	1
neonatal infection	general search	0	0	0	0	0	0	0	0	0	0	0	0	0	0
"sepsis" - include only for newborn or neonatal sepsis	general search	0	3	0	1	4	0	0	0	0	0	0	3	0	11
**QUALITY**															0
quality	general	81	157	122	155	107	23	93	40	72	28	131	189	60	1258
Quality of care	exact	20	29	2	21	15	0	5	12	24	0	33	51	10	222
Quality assurance	exact	0	1	2	25	5	0	2	0	3	0	1	4	5	48
midw*	general	1	8	9	13	9	0	1	0	2	0	19	0	5	67
referral	general	1	7	0	6	11	4	9	1	0	0	1	2	18	60
"family" - looking for family-centred	general	0	0	0	1	0	0	0	0	0	0	0	0	0	1
"respect" - looking for respectful care/disrespect&abuse	general	0	0	0	0	0	0	0	0	0	0	0	0	0	0

Table 4.5: Value of PADs and GFF Grants by country

Country	Total PAD value (US$)	GFF grant component of PAD (US$)	PAD title	Weblink
Burkina Faso	100	20	Health services reinforcement project	https://documents1.worldbank.org/curated/en/570511531107056406/pdf/BURKINA-FASO-PAD-1-06182018.pdf
Cote d'Ivoire	220	20	Strategic purchasing and alignment of resources and knowledge in health project (SPARK-HEALTH)	https://documents1.worldbank.org/curated/en/242801553565658750/pdf/Côte-d-Ivoire-Strategic-Purchasing-and-Alignment-of-Resources-and-Knowledge-in-Health-Project.pdf
Ethiopia	230	60	The health sustainable development goals program for results	https://documents1.worldbank.org/curated/en/228241618346428068/pdf/Disclosable-Restructuring-Paper-Health-Sustainable-Development-Goals-Program-for-Results-P123531.pdf
Kenya	191	40	Transforming health systems for universal care project	https://documents1.worldbank.org/curated/en/215261467995371106/pdf/PAD1694-PAD-P152394-IDA-R2016-0122-1-Box396259B-OUO-9.pdf
Liberia	16	16	Health systems strengthening project (additional credit)	https://documents1.worldbank.org/curated/en/473661488078030716/pdf/Liberia-RP-no-AF-02032017.pdf
Malawi	60	10	Investing in early years for	https://documents1.worldbank.org/curated/en/172701545534083794/pdf/MALAWI-PADf-11302018-636811128679250963.pdf

			growth and projectivity in Malawi project	
Mali	89	10	Mali accelerating progress towards universal health coverage project	https://documents1.worldbank.org/curated/en/382171551732681185/pdf/mali-pad-02282019-636871609042094203.pdf
Nigeria	397	47	Combination of three PADs	
NSHIP	*145*	*20*	*PAD NSHIP-Nigeria State Health Investment Project dated May 2016 (additional credit)*	*https://documents1.worldbank.org/curated/en/380321468179100435/pdf/PAD1741-PJPR-P120977-P157977-IDA-R2016-0125-1-Box396259B-OUO-9.pdf*
Nutrition	*232*	*7*	*PAD HUWE -- Healthcare provision fund project (Huwe project) dated August 2018*	*https://documents1.worldbank.org/curated/en/910491530329489994/pdf/NIGERIA-NUTRITION-PAD-05252018.pdf*
HUWE	*20*	*20*	*PAD Nutrition-Accelerating Nutrition results in Nigeria dated May 2018*	*Document provided by the GFF Secretariat*

Senegal	150	10	Investing in MCA health	https://documents1.worldbank.org/curated/en/469001569808857911/pdf/Senegal-Investing-in-Maternal-Child-and-Adolescent-Health-Project.pdf
Tanzania	300	40	Strengthening primary health care for results program	https://documents1.worldbank.org/curated/en/243871468179947102/pdf/96274-PAD-P152736-IDA-R2015-0121-1-Box391433B-OUO-9.pdf
Uganda	140	30	RMNCH services improvement project	https://documents1.worldbank.org/curated/en/854971471534008736/pdf/PAD-07182016.pdf
